# Measuring receptivity to misinformation at scale on a social media platform

**DOI:** 10.1093/pnasnexus/pgae396

**Published:** 2024-09-10

**Authors:** Christopher K Tokita, Kevin Aslett, William P Godel, Zeve Sanderson, Joshua A Tucker, Jonathan Nagler, Nathaniel Persily, Richard Bonneau

**Affiliations:** Department of Ecology and Evolutionary Biology, Princeton University, Princeton, NJ 08544, USA; Center for Social Media and Politics, New York University, New York, NY 10012, USA; School of Politics, Security, and International Affairs, University of Central Florida, Orlando, FL 32816, USA; Center for Social Media and Politics, New York University, New York, NY 10012, USA; Center for Social Media and Politics, New York University, New York, NY 10012, USA; Center for Social Media and Politics, New York University, New York, NY 10012, USA; Department of Politics, New York University, New York, NY 10012, USA; Center for Social Media and Politics, New York University, New York, NY 10012, USA; Department of Politics, New York University, New York, NY 10012, USA; Stanford University Law School, Stanford University, Palo Alto, CA 94305, USA; Center for Social Media and Politics, New York University, New York, NY 10012, USA; Prescient Design, a Genentech accelerator, New York, NY 10010, USA

## Abstract

Measuring the impact of online misinformation is challenging. Traditional measures, such as user views or shares on social media, are incomplete because not everyone who is exposed to misinformation is equally likely to believe it. To address this issue, we developed a method that combines survey data with observational Twitter data to probabilistically estimate the number of users both exposed to and likely to believe a specific news story. As a proof of concept, we applied this method to 139 viral news articles and find that although false news reaches an audience with diverse political views, users who are both exposed and receptive to believing false news tend to have more extreme ideologies. These receptive users are also more likely to encounter misinformation earlier than those who are unlikely to believe it. This mismatch between overall user exposure and receptive user exposure underscores the limitation of relying solely on exposure or interaction data to measure the impact of misinformation, as well as the challenge of implementing effective interventions. To demonstrate how our approach can address this challenge, we then conducted data-driven simulations of common interventions used by social media platforms. We find that these interventions are only modestly effective at reducing exposure among users likely to believe misinformation, and their effectiveness quickly diminishes unless implemented soon after misinformation’s initial spread. Our paper provides a more precise estimate of misinformation’s impact by focusing on the exposure of users likely to believe it, offering insights for effective mitigation strategies on social media.

**Significance Statement**

As social media platforms grapple with misinformation, our study offers a new approach to measure its spread and impact. By combining survey data with social media data, we estimate not only the number of users exposed to false (and true) news but also the number of users likely to believe these news stories. We find that the impact of misinformation is not evenly distributed, with ideologically extreme users being more likely to see and believe false content, often encountering it before others. Our simulations suggest that current interventions may have limited effectiveness in reducing the exposure of receptive users. These findings highlight the need to consider individual user receptiveness when measuring misinformation’s impact and developing policies to combat its spread.

## Introduction

Exposure to news on social media, whether intentional (1) or incidental (2), has the power to influence beliefs. These beliefs, in turn, can shape perceptions of reality (3, 4) and even fuel social movements (5, 6). While social media has been linked to increased factual political knowledge (7, 8), the proliferation of online misinformation threatens both democracy^a^ and public health,^b^ leading some to deem the presence of an “infodemic” alongside the COVID pandemic (9). In response to this online media environment, researchers from across disciplines (10, 11) are studying the complex interplay between the exposure to (12), belief in (13, 14), and sharing of (15–17) both true and false information online.

However, recent work has yet to unify the measurement of the diffusion of (i.e. sharing and exposure) and actual belief in misinformation. On the one hand, research on misinformation diffusion uses large-scale observational data from social networks to analyze how the spread of misinformation is affected by factors, such as veracity (15), novelty (18), and sentiment (19, 20). Yet, this body of work fails to incorporate a measure of user belief in misinformation, a critical missing component given that not all social media users are equally likely to adopt false beliefs upon exposure to misinformation. On the other hand, research that measures belief in misinformation typically uses survey methods to identify the relationship between belief and individual characteristics, such as ideological congruence (21–24), age (13), cognitive reflection (14, 25), and digital literacy (26). While this body of work reveals key individual-level traits associated with belief in misinformation, it has not been paired with observational social media data to examine how the dynamics of belief play out at scale as misinformation spreads on networked platforms. Even though recent innovations have combined digital trace data with longitudinal survey data (16, 27), these efforts fall short of enabling full-scale evaluations of potential belief across an entire social media platform. As a result, we lack an estimate of the scale of receptivity to misinformation among those exposed—that is, a measure of the number of users who are likely to believe the misinformation that they see—limiting our understanding of the real impacts of misinformation circulating on social media.

Likewise, without the ability to measure potential receptivity to misinformation at scale, we are not able to fully evaluate the effectiveness of interventions designed to reduce the impact of misinformation on social media users. Recently, social media platforms have used various strategies to limit the spread of misinformation (28), such as labeling questionable articles with fact-check labels (29, 30), making them more difficult to share (17, 31), or simply reducing their visibility on users’ feeds (32). Despite the rapid rise of these platform-level interventions (33) and the subsequent debate over whether they are worthwhile (34), we lack a way to measure how these interventions might ultimately change misinformation exposure among the users most likely to believe it (i.e. users who are receptive to believing a particular piece of misinformation to be true). While recent work has provided insight into how interventions could reduce the likelihood of users sharing misinformation (35), these insights stop short of understanding how such interventions alter exposure among users who are receptive to believing false stories. Research that does aim to measure the impact of interventions on belief in misinformation typically focus on individual-level effects (17, 36), overlooking the broader platform-wide dynamics that occur when misinformation diffuses through a social network, where a share can potentially expose thousands of other users. Therefore, accurately measuring the scale of potential belief, while also accounting for the spread of (mis)information on social media platforms, is crucial for a full assessment of the effectiveness of interventions.

Aiming to bridge these two different approaches to researching misinformation, we introduce a method to estimate user receptivity at the scale of a social media platform. Our approach combines individual-level surveys assessing belief in news stories with observational data on the spread of those same stories across a social media network. By combining surveys with large-scale platform data, our method parallels previous work estimating the effect of information exposure (37), though here we use a different—yet potentially complementary—measurement of impact. As a case study, we estimate the number of users receptive to believing 139 highly popular US news articles, 102 of which were true and 37 of which were false or misleading,^c^ as well as the spread of those articles across Twitter from the time of their initial publication. Our approach combines (i) large-scale Twitter data tracking the spread of these articles and (ii) real-time surveys measuring how likely ordinary Americans are to believe the articles’ contents. Because we cannot directly measure user belief—that is, we cannot causally say that a particular user saw an article and consequently changed their beliefs—we focus on the exposure of *receptive users*: those who both see an article and are predicted to believe it, estimated here as a function of an exposed user’s political ideology.

Importantly, we note that our study serves as a proof of concept, aiming to demonstrate the insights that can be gained from this new approach to measuring the impact of misinformation. We acknowledge that our methodology relies on simplifying assumptions about user exposure, which may not fully capture the complexities of exposure and belief on social media platforms. However, by presenting this initial framework, we lay the groundwork for future research to refine and build upon our methodology, including combining this approach with other innovative methods for measuring susceptibility among users (38).

Using this new approach, we show that while users across the ideological spectrum encounter misinformation, the exposed users who are receptive to misinformation are mostly on the political extremes. In contrast, users who are exposed and receptive to true news in our sample are more ideologically moderate. This discrepancy underscores the need to focus on users who are not just exposed but are also receptive to misinformation. Crucially, those who see both true and false news earliest after publication are also the users most likely to be receptive to believing it to be true, highlighting that misinformation can potentially be believed by millions of users in only a few hours. Taken together, our findings build upon previous research showing the swift propagation of false news (15) by demonstrating that the impact on users’ beliefs occurs mainly among those at the political extremes and likely transpires early during the spread of misinformation. Because these findings highlight the potential challenge social media platforms may face when trying to mitigate misinformation, we then use our exposure and receptivity data in data-driven simulations to evaluate the effectiveness of various misinformation interventions. These simulations reveal that interventions are only modestly effective at reducing the exposure of users receptive to misinformation, with their effectiveness further declining unless implemented within a few hours of the initial sharing of misinformation on social media.

## Results

Using a preregistered sampling strategy (13), we collected a dataset of 139 top-trending articles and subsequently measured their dissemination on Twitter. Our goal was to estimate, for each article, the number of Twitter users exposed to the article and the subset of these users that were most likely to believe each article to be true. We call this second subset *receptive users* because we estimate that they are likely to believe an article to be true, though to be clear we cannot causally confirm that the beliefs of individual Twitter users have changed. We estimated the number of *exposed* and *receptive* users by combining two types of data. First, we used Twitter data to identify which users on Twitter were potentially exposed to each of our 139 articles; we also estimated each potentially exposed user’s ideological placement on a liberal-conservative scale. This process yielded $x_{ij}$, the number of users within each ideological category *i* that were exposed to article *j*. Second, to determine the likelihood that these exposed users would believe an article to be true, we deployed surveys in real-time as each article spread online. These surveys asked Americans who are habitual internet users—a demographic proxy for social media users—to classify the article as true/false and provide demographic information, including ideology. From this survey data, we calculated $p_{ij}$, the proportion of individuals within each ideological category *i* that believed article *j* to be true. With these two estimates for each article *j*—the number of exposed users by ideology $x_{ij}$ and their probability of belief by ideology $p_{ij}$—we could calculate the number of Twitter users exposed and receptive to believing the article to be true, $r_{j}$:


$$r_{\;j}=\sum_{i}x_{ij}p_{ij}$$

On each weekday between 2019 November and 2020 February, we identified 139 articles that were the most popular daily articles from a mix of mainstream and fringe news sources across the ideological spectrum. Our research team did not select these articles—a process known to undermine external validity in previous studies (39)—nor did we simply take a random sample of all news articles published each day. Instead, our preregistered sampling method identified the most popular article from each of five news streams,^d^ creating a sample of *highly popular* articles that spanned source quality and ideological lean (see Materials and methods for more information about the article sampling strategy, which is also used in (40, 41)).

To establish the veracity of the articles in real time, we sent each article to six professional fact-checkers within 48 hours of publication. The fact-checkers independently rated each article as “true” or “false/misleading,” and we then used the modal rating from the fact-checkers to label an article as “true” or “false/misleading” in our dataset (see Materials and methods section for details about article collection and fact-checking). In the end, fact-checkers rated 102 articles as “true” and 37 articles as “false/misleading.”^e^

Concurrently with this fact-checking process, we also tracked the spread of each article on Twitter. We collected all tweets and Twitter users that shared an article’s URL within one week of its publication. We also collected the friend and follower lists of users who shared an article to identify all users potentially exposed to the article on Twitter. Because Twitter does not release data on which tweets were seen by a given user, we make the simplifying assumption that all users who follow someone who tweeted an article URL link were eventually exposed to that article (see Materials and methods for details on how we estimate exposure time). Although this assumption may not fully capture the complexities of actual exposure on Twitter, it enables us to estimate an upper bound of “potential exposure” without relying on arbitrary assumptions about the platform’s proprietary news feed algorithm. By using “potential exposure” as a proxy for actual exposure, we prioritize simplicity and interpretability in our proof-of-concept approach. Despite this simplifying exposure assumption, our approach still provides valuable insights into the relative patterns of exposure and receptivity across different user groups, which are likely to hold even if the actual exposure levels differ from our estimates. As is the convention in the literature (27), we refer to these potential exposures simply as “exposures” for the remainder of the manuscript.

Because ideology is a key predictor of belief in a particular article (13, 21–24), we needed to assign estimates of ideology (hereafter “ideology scores”) to all exposed Twitter users so that we could in turn estimate how many users were receptive to believing an article was true. Using the method from (42), we calculated the ideology of all Twitter users who shared one of the articles in our study, as well as their followers and friends. If we did not have enough information to directly calculate the ideology of an article sharer using the method in (42), we calculated their ideology as the mean of their friends’ ideology. Finally, to fill in missing ideology scores among a sharer’s followers, we used a Bayesian approach that used the known ideology scores among their followers to infer what the rest of their followers’ ideologies could reasonably be (see Materials and methods for more detail).

To estimate which exposed Twitter users were most likely to believe each article, we fielded surveys among everyday Americans within 48 hours of each article’s publication. Each survey asked a panel of roughly 90 respondents recruited by Qualtrics to evaluate an article’s veracity. These panels were made up of habitual internet users and were balanced on age, gender, education, and ideology. Importantly, because each survey was conducted concurrently with an article’s circulation online, each panel’s response provided a reasonable proxy for how social media users would interpret a particular article. Survey respondents were asked to read three randomly selected articles from the day’s five trending news articles and then rate each as “true,” “false/misleading,” or “could not determine.” Respondents also provided demographic information, allowing us to correlate the likelihood of believing an article with self-reported ideology. In total, 5,072 unique respondents evaluated the 139 articles in our dataset, resulting in 13,582 individual article evaluations. Consistent with previous research (22, 23), we found that the most prominent demographic predictor of a respondent’s belief in the article was alignment between their own ideology and ideological slant of the article (see Supplementary Material, Text Figure S1 and (13) for details).

Finally, by combining our estimates of user exposure and ideology from the social media data with our estimates of belief probability from the surveys, we calculated the number of users who were exposed and *receptive* to each article. For a each article, this calculation involved counting the number of users within each ideological category and then multiplying each count by the corresponding belief rate among survey respondents in the same ideological category. Crucially, our estimates are based on the actual reactions of internet-frequenting Americans to each specific article and do not depend on how we rated the veracity and slant of an article. To illustrate our general approach, suppose we estimated that 100 liberal and 100 conservative users were exposed to an article. If we know from the surveys that 40% of liberals and 10% of conservatives believed the article to be true, our method would then estimate that 40 liberal users and 10 conservative users were likely receptive to believing the article to be true. Our approach makes a simplifying assumption that a user’s receptivity depends only on their ideology. We make this assumption because ideology has been shown to be a strong predictor of belief in news veracity (13, 21–24) and can be readily estimated from Twitter friend–follower networks (42).

We have three primary motivations for bringing together the measurement of exposure and receptivity to misinformation. First, we examine whether the overall pattern of user exposure mirrors that of the users most likely to believe a piece of misinformation to be true. While many studies assume that user exposure is a proxy for the impact of misinformation (12, 43, 44), they do not account for variation in user receptivity to believing false news stories to be true. Therefore, it is important to understand whether researchers can safely assume that measuring general user exposure accurately captures who is believing misinformation to be true. Second, we measure how quickly exposure accumulates among users likely to believe misinformation once an article begins circulating on social media, as this rate is crucial for the success of any efforts to mitigate belief in misinformation. Third, using simulations, our methods allow us to assess the potential effectiveness of common interventions employed by social media platforms to blunt exposure to misinformation.

### Receptive users exposed to misinformation are more ideological extreme than nonreceptive users

**Fig. 1.**
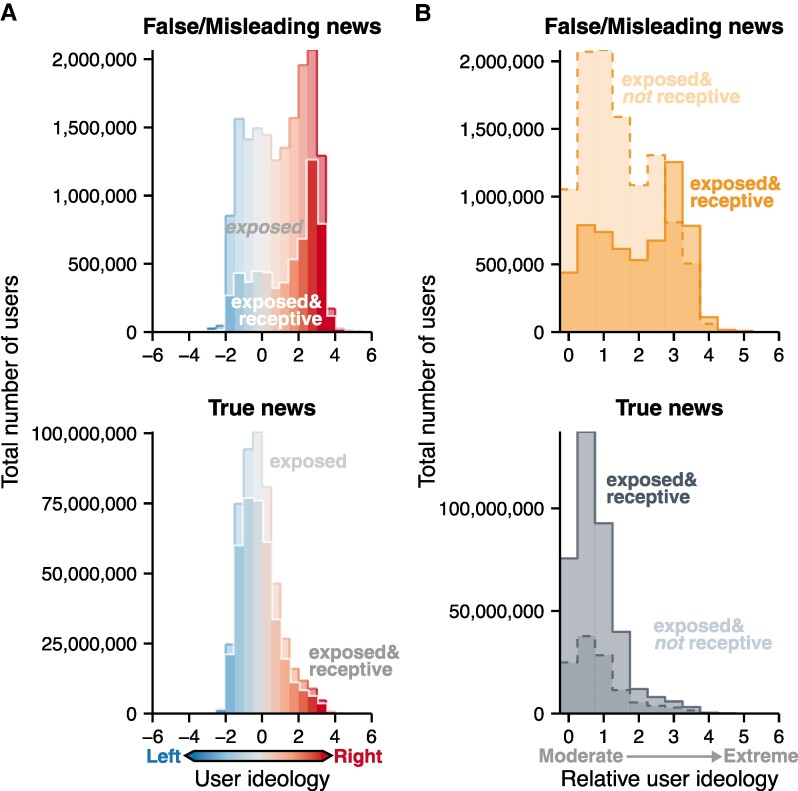
Estimated number of users exposed and receptive to top-trending news articles on Twitter. A) Estimates for the total number of users who were potentially exposed to the true and false/misleading news articles in our dataset. For user ideology, negative values indicate left-leaning ideology and positive values indicate right-leaning ideology. B) Estimates for ideological extremity among the users who were potentially exposed to news articles in our dataset. For the relative user ideology, low values indicate users with slightly left-leaning or right-leaning ideology, while large values indicate extreme left-leaning or right-leaning ideology.

We find that tens of millions of unique Twitter users were potentially exposed to the articles in our dataset (Figure 1A). Among these articles, 37 were rated as false/misleading by professional fact-checkers. These 37 articles, in turn, generated over 16.5 million potential instances of unique user exposure. Notably, this included over 5.8 million potential instances where users were exposed and receptive to incorrectly believing the misinformation to be true. On the other hand, the 102 news articles that were rated as true by professional fact-checkers generated 492 million potential instances of unique user exposure. Of these, 375 million instances involved users who were exposed and receptive to correctly believing the articles are true. Therefore, our estimates suggest that, compared to false news articles, true news articles in our study were seen by nearly two orders of magnitude more Twitter users receptive to believing their content.

Using ideology estimates of Twitter users derived from their follower networks, we find striking differences between the pattern of exposure to misinformation between users receptive to believing it and those who are not (Figure 1B). Compared with true news, misinformation was shared by Twitter users with more extreme ideologies (Figure S2). Despite this, the majority of users exposed to these false/misleading articles were not extreme and were actually quite ideologically diverse. However, among these exposed users, those receptive to believing the misinformation to be true tended to have more extreme ideologies, while those who were not receptive had far more moderate ideologies. In contrast, for true news articles, the pattern of exposure did not differ between users who were receptive to believing them and those who were not (Figure 1B). Across the ideological spectrum, users saw and were similarly likely to believe the true news articles, resulting in ideologically moderate users making up the majority of both the receptive and nonreceptive groups. Even when normalizing for article virality, we still observe this same difference in receptivity between true and false news (Figure S3), indicating that ideologically extreme users are more likely to see and believe misinformation regardless of how widely an article spreads. This key role played by more extreme users in misinformation exposure and belief adds to recent research on the dynamics of political acrophily on social media, the tendency for social media users to prefer and interact with in-group members who hold more extreme political views (45).

**Fig. 2.**
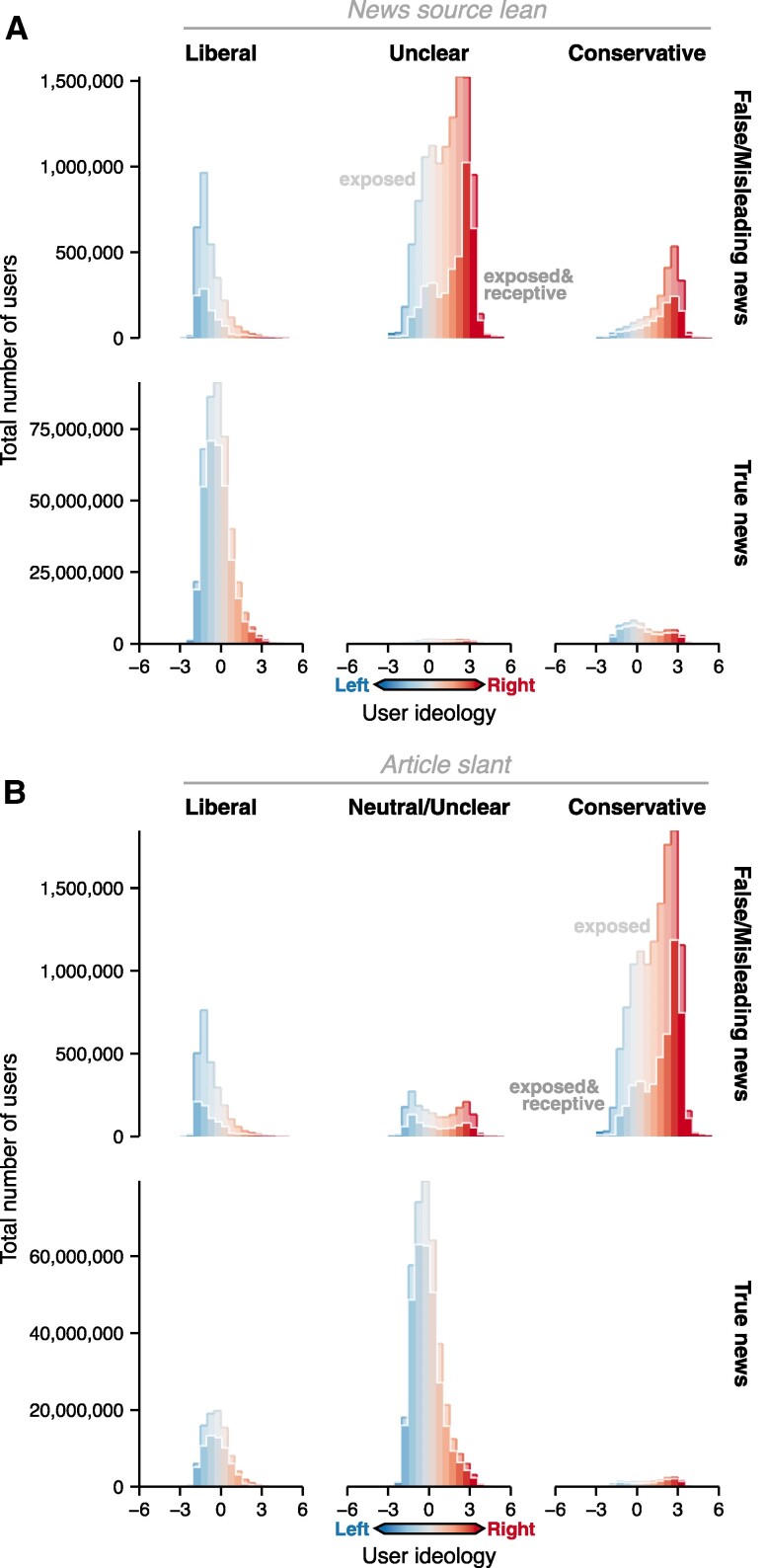
Total number of users exposed and receptive to articles, broken out by A) news source lean and B) article slant. For user ideology, negative values indicate left-leaning ideology and positive values indicate right-leaning ideology.

When categorizing articles by their ideological slant, we see that ideologically extreme users are disproportionately likely to see and believe articles that have content that aligns with their own ideology (Figure 2). Right-leaning users were disproportionately likely to both see and be receptive to believing articles with a right-leaning slant, while left-leaning users were disproportionately likely to see and be receptive to believing articles with left-leaning slant (Figure 2B and Figure S4). This tendency, paired with a mix of left- and right-leaning misinformation, resulted in most exposure among users receptive to misinformation occurring among those at the ideological extremes. Conversely, for true news, most user exposure was generated by articles that had a neutral slant, and the exposure pattern for users receptive to believing the articles generally matched the overall pattern of user exposure across the ideological spectrum.

These findings underscore an important dynamic: general measures of misinformation exposure can obscure the more nuanced and significant pattern among the users most susceptible to believe it. Therefore, to properly design and time interventions aimed at lowering the impact of misinformation, we must understand when users receptive to believing these are articles are exposed, rather than relying solely on measurements of general user exposure. In the next section, we explore how exposure unfolds differently over time for users receptive to believing misinformation when compared against the general user base.

### Exposure to misinformation among receptive users quickly accumulates over the first 48 hours

While we have characterized the ideological composition of exposed users who are receptive to believing true or false/misleading news, it is also important to understand how this exposure unfolds over time as an article is circulating through social media. Therefore, for each news article, we analyzed the exposure of users—both those who were receptive to believing the articles and those who were not—over the first 48 hours after a news URL is first shared, a critical period in which most sharing occurs (15).

**Fig. 3.**
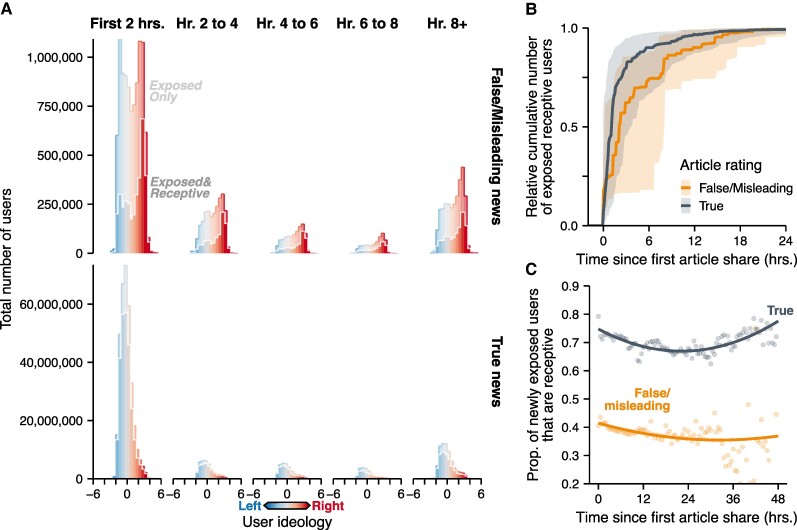
A) The number of users exposed and receptive to news articles over the first 24 hours of article sharing. For user ideology, negative values indicate left-leaning ideology and positive values indicate right-leaning ideology. B) The cumulative number of users who are exposed and receptive (median with interquartile range) over time, normalized across articles. C) Proportion of newly exposed users that are receptive to believing an article to be true. The lines are the best-fit Bayesian regression models applied to all raw Tweet data. For ease of visualization, the points show the binned mean of tweets aggregated within 30-minute intervals.

We find that the majority of news article exposure among the users most likely to believe them happens within the 6 hours after an article URL is first shared on Twitter (Figure 3A). By hour six, the average true news article reaches 78.2% of its total potential receptive audience over the initial two-week period after publication, crossing 50% within 2 hours. Similarly, the average false/misleading news article reaches 60.8% of its total potential receptive audience within 6 hours, with the average article crossing 50% within 3 hours. (Figure 3B). When comparing the time it takes to reach 50% of cumulative exposure among users receptive to believe the content to be true, we did not find a significant difference in the rate at which true and false/misleading news accumulate exposure among users receptive to believing them (t(55.4)=−1.154,P=0.25). Overall, these findings highlight the speed at which information—both legitimate and not—can spread and impact the beliefs of millions of social media users.

We find that news articles in our sample have their highest rate of exposure among users receptive to believing them in the hours immediately after publication. During the first hour after a news URL is first shared on Twitter, an average of 76.1% of the exposed users were receptive to believing a true news article, but this rate drops significantly to 66.1% by the 24th hour (Figure 3C; Mann–Whitney U=6,567,052, $n_{1}=11,819$, $n_{24}=1,100$, P<0.001). Conversely, for false/misleading news articles, initially only 42.8% of exposed users are receptive to believing them to be true, dropping further to just 37.7% within 24 hours (Figure 3C; Mann–Whitney U=286,842, $n_{1}=3,633$, $n_{24}=352$, P<0.001). Interestingly, by the 48th hour, we observe a significant rebound in the frequency of receptive users among those newly exposed users to true news (Mann–Whitney *U* = 23,014, $n_{24}=1,100$, $n_{48}=60$, P=0.008), but we do not see a rebound among false/misleading news (Mann–Whitney U=512.5, $n_{24}=352$, $n_{48}=10$, P=0.42). However, the data is relatively sparse by hour 48, which may limit the ability to generalize the observed rebound effect for true news. It is important to note that our surveys of user belief in articles were conducted immediately after publication (see Materials and methods), so we did not track shifts in baseline belief rates among users as discussion of an article spreads. Instead, in our estimates, the observed decrease over time in the proportion of users receptive to believing an article is driven entirely by the shifting composition of the audience: users with ideologies that are less inclined to believe an article tend to get exposed later.

**Fig. 4.**
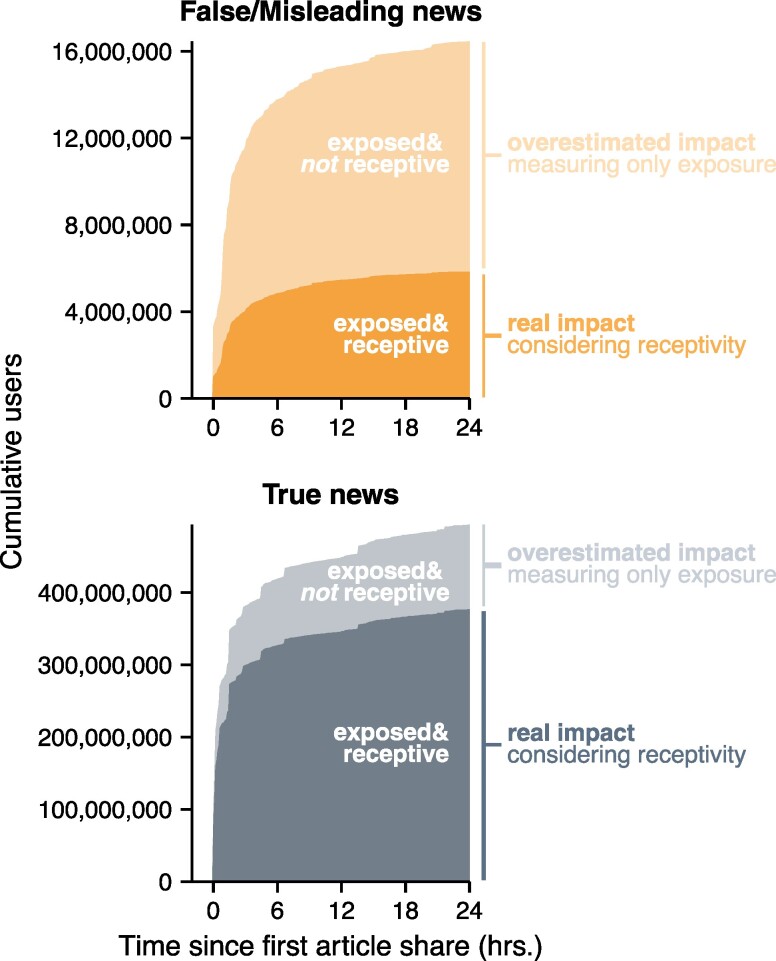
Considering user receptivity gives a very different estimate of the impact of misinformation. Cumulative exposed users over time across all false/misleading news articles (n=37) and true news articles (n=102) in our dataset, broken out by whether the exposed users were receptive to believing the news article to be true.

In contrast to research finding that misinformation spreads faster than true information (15), we find that true news articles are seen by a receptive audience faster than false/misleading news. In the initial hours after an article URL is shared online, true news articles are seen by far more users receptive to believing them when compared against false/misleading news articles (Figures 3A and 4). This gap widens substantially over the course of a week, ending with nearly two orders of magnitude more users being exposed and receptive true news articles (Figure 1A). By considering not just exposure but also individual users’ receptivity to each news article, we reveal that the real impact of misinformation is likely far less than what baseline exposure alone would suggest (Figure 4). This pattern may be partially driven by the news source itself: articles from fringe news sources are seen by fewer users (Figure S5) and are less likely to be believed by exposed users than articles published by mainstream sources (Figure S6). Thus, true news articles, typically published by mainstream sources, have a larger audience that is more likely to believe their contents, allowing true news articles to generally accumulate exposure among a receptive audience faster than false/misleading articles. Overall, these patterns underscore that the dynamics of belief—as implied by our measurement of users who are both exposed *and* receptive to believing an article—cannot be simply extrapolated from the pattern of general user exposure but must also account for individual and network dynamics, such as the quality of the news source and the ideology of the exposed audience.

### Common social-media platform interventions are largely ineffective at preventing receptive users from being exposed to misinformation

Social media platforms often deploy interventions in an attempt to stop the spread and impact of misinformation. To evaluate the potential effectiveness of such interventions, we conducted data-driven simulations of common platform-level interventions and estimated how they reduce exposure among users receptive to believing misinformation. We focused our simulations on the articles in our dataset that were labeled as false/misleading by professional fact-checkers, mirroring platform policies that only act on articles that have been evaluated as false by external reviewers (46–49).

We explored three simple, commonly used interventions (50) (see Materials and methods): sharing friction, fact-check labeling, and visibility reduction (e.g. downranking). We ran simulations in which we set individual-level effects of interventions and examined how they changed misinformation exposure among all users and among users receptive to believing it. With the exception of visibility reduction, our estimates of the individual-level effects of interventions were based on results from other studies. For sharing friction—adding extra steps to the retweet process for tweets sharing flagged material—we assumed that they made an individual 75% less likely to retweet a flagged article (17). For fact-check labeling, we assumed that they made an individual 25% less likely to retweet (17) and 17% less likely to be receptive to (36) a flagged article. Finally, for visibility reduction—making tweets containing a flagged news article less likely to appear in other user’s feeds—we assumed a light and heavy version of the intervention: the interventions made misinformation tweets 25% (light) or 75% (heavy) less likely to appear in other user’s timelines. Unlike the sharing-focused interventions, the rates for visibility reduction interventions were selected for ease of comparison and were not based on findings from studies, as there have not been, to our knowledge, any publicly available studies.

We also considered the timing of intervention deployment, focusing on the delay $t_{\mathrm{int}}$ between an article’s appearance and the start of an intervention. When considering using an intervention on a potential piece of misinformation, a social media platform will commonly use professional fact-checkers to first determine the veracity of the article (51). While external fact-checkers helps ensure that interventions are only used on false or misleading content, this approach is accompanied by a tradeoff: it takes time for fact-checkers to verify a news article, allowing the misinformation to circulate freely for some time. Therefore, our simulations estimated the effect of review time on intervention efficacy. We assumed that an intervention is deployed $t_{\mathrm{int}}$ hours after a piece of misinformation first appears on the platform, where $t_{\mathrm{int}}$ represents the review delay. We also simulated an ideal case where interventions can be immediately deployed on a piece of misinformation ($t_{\mathrm{int}}=0$), as might be possible with the use of artificial intelligence that can instantly flag questionable content.

**Fig. 5.**
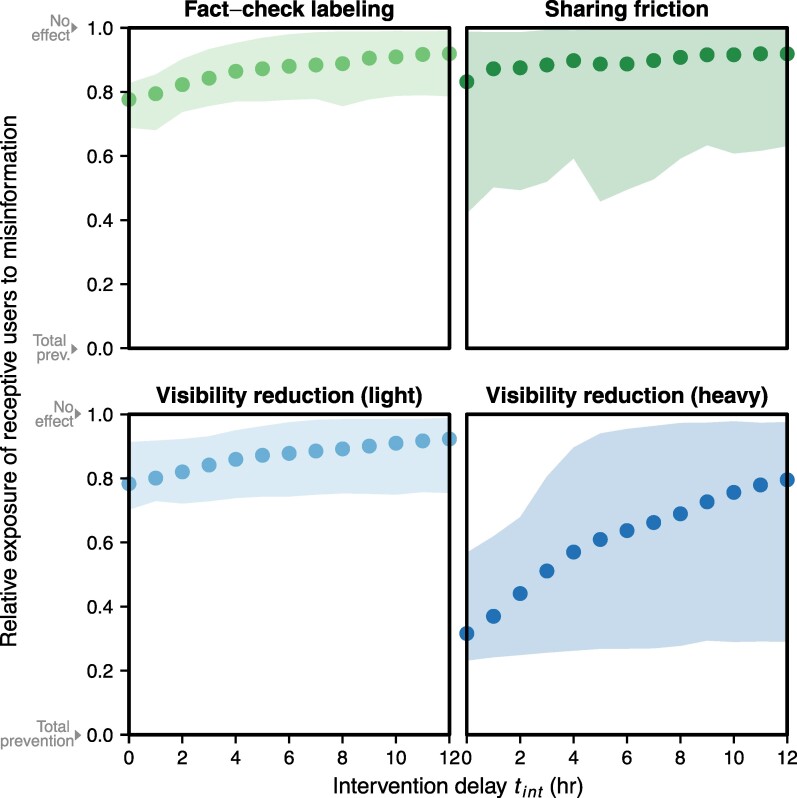
Simulating how intervention method and timing reduce misinformation exposure among the users most likely to believe it. Intervention delay $t_{\mathrm{int}}$ is the number of hours between an article’s publication and the deployment of an intervention. Fact-check labeling decreases the probability of retweets by 25% and the probability of individual receptivity by 17%, while sharing friction—adding extra steps to the retweet process for tweets sharing flagged material—decreases the probability of retweets by 75%. Visibility reduction is an intervention in which a tweet containing a flagged news article becomes 25% (light) or 75% (heavy) less likely to appear in other users’ feeds. Points indicate the mean of data-driven simulations across all false news articles in our dataset, with each ribbon encompassing 90% of all simulation outcomes. We simulated each article 10 times for a given intervention type and delay.

Even when assuming that interventions can be deployed immediately, we find that simple interventions aimed at reducing the sharing or visibility of false news articles achieve mixed success in lowering exposure among all users (Figure S7) and particularly among users receptive to believing the misinformation (Figure 5). Attempts to slow the sharing of misinformation generally have a very modest effect on the overall number of users who are exposed and receptive to believing the misinformation to be true. Even assuming that it reduces the likelihood of individual retweeting by 75%, sharing friction only reduces exposure among users receptive to believing misinformation by an average of up to 16.5%. Similarly, despite reducing the likelihood of individual retweeting by 25% and the likelihood of individual belief by 17%, fact-check labels reduce exposure among users receptive to believing misinformation by an average of up to 22.4%. The limited effectiveness of these sharing-focused interventions is partly because many of these articles are initially shared by accounts with large follower counts, ensuring that many still see the tweet even with reduced retweeting. In contrast, visibility-focused interventions—where Twitter makes flagged misinformation tweets less likely to appear in other users’ feeds—are more effective. Assuming that users would be 25 or 75% less likely to see a tweet sharing misinformation, visibility reduction decreased exposure among users receptive to believing misinformation by an average of up to 21.7% and 68.2%, respectively. Unlike sharing-focused interventions that only affect retweets, visibility-interventions are more impactful because they decrease the likelihood that a user sees both original shares of an article URL and rewteets.

However, the effectiveness of these fact-checker-backed interventions depends on how quickly they can be deployed. The longer it takes for professional fact-checkers to verify an article, or for a social media company to act after the fact-check, the less an intervention can reduce misinformation exposure among users receptive to believing it. For example, if Twitter manages to fact-check questionable articles within 1 hour of their first appearance on the platform, we estimate that a heavy visibility reduction intervention can reduce the exposure of users receptive to misinformation by an average of 60% or more—a number that could easily result in millions fewer users believing false or misleading news articles on social media. Conversely, if it takes over 10 hours to fact-check articles and deploy interventions, we estimate that heavy visibility reduction interventions can reduce the exposure of users receptive to misinformation by an average of 25% or less. Given the infrastructure and coordination required for third-party fact-checking, the timing of this intervention method is a significant consideration.

## Discussion

Our study introduces a new approach to estimate user receptivity to believing the veracity of highly popular news articles at a platform level, focusing on both the exposure of users to content on a social media platform *and* the likelihood that users will believe that content to be true. We have combined two common approaches to studying misinformation: the analysis of social media data to measure an article’s spread and survey-based studies to measure individual-level predictors of belief in the veracity of news stories. Our study serves as a proof of concept demonstrating the insights that can be gained from a more precise measurement of misinformation impact that incorporates both spread and likelihood of belief. In some contexts, such as the uptake of the COVID vaccine, the impact of misinformation can be measured through behavioral outcomes, drawing on the approach in (37). In other contexts, such as news about political events, there are no clear behaviors related to information exposure, so the impact of misinformation can be measured by receptivity, using the methods presented here. As with most models of this nature, our methodology relies on simplifying assumptions. In particular, our assumptions may not fully capture the nuances of exposure on social media platforms, especially given the opaque algorithms platforms use to curate user feeds. However, by presenting this initial approach, we aim to pave the way forward for misinformation research, offering a foundation that can be refined and expanded to better understand the impact of news—both false and true—as it spreads through social media platforms.

Despite its limitations, we believe this novel approach—this first attempt at combining social media data and survey data to create a more precise measurement of the impact of online misinformation—has promise not only for researchers but also for social media platforms. It provides a tool to measure the complex interplay of article exposure, receptivity to believing articles to be true, and article sharing across the entire social media platform. This article-based approach can also be combined with recent work that uses Twitter data to examine user susceptibility at the source level (38). Importantly, our approach also allows for data-driven simulations of interventions, offering insight into the effectiveness of various platform policies aimed at minimizing the impact of misinformation. Future research could refine our approach to more precisely measure exposure or incorporate additional predictors of individual belief, such as cognitive reflection (52) or information literacy (53).

Beyond demonstrating a new method for estimating news exposure and receptivity to believing a news article to be true at the population level, our study reveals the divergence in exposure patterns between the general user base of on a platform and the population of users who are receptive to believing an article to be true. This distinction is particularly relevant to researchers measuring the impact of online misinformation. While current approaches focus on the patterns of sharing (12, 15, 35, 54) or general user exposure (12, 43, 44), they overlook the individual predictors of belief among those exposed. Our study shows that misinformation can expose a broad audience despite being shared by an ideologically extreme set of users. However, the political slant of these false articles greatly skewed which exposed users were receptive to incorrectly believing the misinformation to be true, since users were far more likely to be receptive to misinformation that aligned with their political leaning. In contrast, true news did not have this disconnect between exposure among all users and exposure among users likely to correctly believe the content to be true. Additionally, we found that the receptiveness of an article’s audience evolved as it spread: users who were most likely to believe an article tended to see it earlier than more skeptical users. Collectively, our findings demonstrate that those who see an article can look quite different from those who ultimately believe it to be true. This nuanced insight could only be generated by simultaneously considering who is likely to see an article, who is likely to believe the article to be true, the article’s veracity, and the article’s ideological slant.

Of course, the magnitude of certain findings, such as the amount of cross-ideological exposure, depends on the assumptions of our simulations and our article selection method. Of particular importance, we assumed that all followers of a given user are eventually exposed, which we know is not the case in reality. For instance, news feed ranking algorithms probably make users more likely to see content that aligns with their ideology (55). Still, even if news feed algorithms result in less cross-ideological exposure, our fundamental results would hold: the overall pattern of exposure among all users would still obscure the more critical pattern of which users were most likely to believe article content, since users are more likely to believe content that aligns with one’s own ideology (13, 21–24). In fact, if news feed algorithms did reduce cross-ideological exposure, we would expect misinformation to increasingly reach and be believed by users on the ideological extremes rather than those with moderate ideologies. Similarly, our article selection method generally selects for viral articles, which may not be representative of most news articles that have much smaller circulation. For example, while a viral political article might be seen by many users across the ideological spectrum, the median political news article might remain within a specific partisan circle and therefore have far less cross-ideological exposure.

Our findings challenge the prevailing notion that false news spreads faster and wider on Twitter than true news (15). We instead estimated that true news articles in our sample accumulated far more exposure among users likely to believe them in the initial hours after first appearing on Twitter. This was primarily because true news tends to be seen by more users and believed at a higher rate. Thus, our findings align with previous research finding that the impact of fake news on social media might be overstated (12, 16, 56). However, since our study focused on a limited number of top-trending news articles, future research will need to see if this pattern holds across a broader set of news articles. Nonetheless, like other research (12, 56–58), we did find that misinformation sharing and receptivity was concentrated among users with more extreme ideologies. This finding underscores concerns that misinformation could reinforce political divisions (59) and further sort social networks along political lines (60).

Our results also highlight the asymmetric nature of Twitter’s underlying social network structure, which may contribute to the observed disparities in how articles are seen and believed. Theoretical models predict that moderate users should serve as connectors who bridge the ideological divide in social networks (60). Our results partially supported this prediction, finding that moderate users did indeed have more ideologically diverse follower networks. However, we also found that center-right users had more diverse social connections than their center-left counterparts, allowing articles shared in right-leaning circles to gain a more diverse audience than those shared in left-leaning circles. This structural asymmetry in the social network might explain why far right content seems to receive amplification on social media (61). Future research will need to delve deeper into the ideological organization of online social networks, but our findings suggest that this structural bias could drive major differences in how certain articles gain traction online.

Our simulations suggest that common interventions for reducing the impact of misinformation may be less effective than planned, particularly when it takes hours to identify, verify, and target a piece of misinformation. Due to the fact that exposure among users likely to believe misinformation quickly builds in the first few hours of circulation on Twitter, our simulations showed that interventions can only be effective if they are implemented within a few hours of the URL first being posted. This urgency likely extends to other platforms as well, suggesting a very consequential consideration for how platforms plan and deploy interventions against misinformation. However, since our findings are based on a sample of popular articles, future research should explore the possibility that interventions may be more effective against less popular, slower-spreading content. Nonetheless, research suggests that social media platforms could improve the effectiveness of interventions by combining multiple interventions (35) or instead using psychological nudges (14, 62), such as user interface cues that encourage users to focus on the accuracy of the article, but these methods are still very time-sensitive. The largest gain in effectiveness could come from methods that speed up the article verification process, offering faster turn around than traditional fact-checking—methods such as machine learning (63, 64) or crowd-sourcing (63, 65, 66). Alternatively, prebunking might also prove effective, as it does not require classifying individual articles but instead aims to preemptively inoculate users against emerging misinformation campaigns (67).

Given the importance of speed for intervention effectiveness, the use of language and multimodal models for content moderation may be a promising area for further research (68–72). While their application to moderating content “in the wild” likely requires more rigorous testing, particularly for under-resourced natural languages (73), the advances in these models offer the possibility to augment the speed, scale, and precision of moderation systems, especially when used in conjunction with human moderators for edge cases. Indeed, major LLM developers, such as Anthropic^f^ and OpenAI,^g^ already offer moderation endpoints for their state-of-the-art models, enabling scalable classification of harmful text that can be trained on a platform’s specific policies (74). While likely not a panacea for content moderation, AI-based systems introduce the possibility of addressing the temporal challenges highlighted in this paper.

In addition to demonstrating that implementation speed is key for effective misinformation interventions, we also found that interventions aimed at reducing the general visibility of misinformation (e.g. downranking) are more effective than other methods. In our simulations, we cautiously assumed that visibility-focused interventions could reduce the likelihood of misinformation showing up in users’ feeds by up to 75%. This was a rather conservative assumption given that aggressive downranking can virtually eliminate the presence of misinformation tweets. Even with this conservative estimate, we still found that downranking far out performed fact-check labeling and sharing friction. If our simulations had used more aggressive assumptions for downranking (e.g. 90% visibility reduction), the difference in effectiveness between visibility-focused and sharing-focused interventions would likely be even more pronounced. Of course, it is important to note that any reliance on downranking raises crucial questions about the criteria for determining which articles should be downranked, a complex issue that is beyond the scope of this paper.

Our study has three main limitations. First, rather than directly measuring user exposure on Twitter, we had to rely on simulations with simplifying assumptions to estimate when a user might reasonably be exposed to an article. While not ideal, this approach was necessary due to a significant challenge in collecting data from major social media platforms like Twitter: at the time of our data collection, Twitter’s API did not provide information on user exposure. Second, our approach used inferred ideology as the only individual-level predictor of a user’s receptivity to believing a particular article to be true. While other characteristics, such as cognitive reflection (75), also influence receptivity to misinformation, we are not able to infer these traits from observed digital trace data. Fortunately, previous work shows that ideology is the strongest predictor of belief in misinformation (13). However, by using ideology scores inferred from user Twitter data, we may be introducing error in our estimates. These inferred ideology scores inherently contain some noise, and translating continuous scores into categorical ideology bins can add more noise. Although a user’s inferred ideology generally aligns with their self-reported ideology category, particularly with regard to their position on the general left-right political spectrum, some users still get misclassified. This misclassification of ideology leads to incorrect assumptions about a user’s likelihood of believing a news article to be true. As a result, these errors can propagate and skew our top-level estimates of how many users are receptive to misinformation and news. Third, and most importantly, our approach may not translate easily to other popular social media platforms. Twitter is a large public platform with a unique network structure (76) and a user base that is not representative of the broader American public (77). While our approach relied on a well-established method for inferring user ideology on Twitter (42, 78)—and, to some extent, Facebook (58)—it may not be transferable to other popular platforms, such as YouTube and TikTok, that have different user dynamics and network structures.

Future work can build on the modeling and data collection methods we have pioneered in this paper. Although we used a systematic data collection method to ensure that the articles in our study were top-trending articles from a cross-section of news outlets, the daily set of five articles is still a limited snapshot of trending news online. Moreover, we used simplistic assumptions—e.g. all followers are eventually exposed—as a first approach, but future work could implement more realistic assumptions grounded in data, including data that may become available to researchers through the European Union’s Digital Services Act (DSA). For example, an extension of our method could use agent-based models to compare the spread of true and false news side-by-side under different intervention scenarios. This would enable a more comprehensive assessment of the costs and benefits of certain interventions—e.g. one could quantify the reduction of misinformation exposure among users likely to believe it and weigh it against the amount of true news that is inadvertently impacted by interventions. Additionally, future work should refine how user ideologies are estimated and translated into belief rates. For instance, adjusting the bins that translate continuous ideology scores into survey-like ideology categories could reduce errors. While our results show that misclassifying some users into adjacent categories—e.g. “Conservative” instead of “Slightly Conservative”—does not greatly impact overall findings, reducing this source of error would strengthen the estimates of user receptivity to misinformation on social media.

## Methods and materials

In this section, we detail the data and methods used in this paper. In the first three subsections below, we describe how we selected a dataset of 139 news articles and then collected the survey data and separate Twitter data for each article. Next, we explain how we constructed the retweet networks within the Twitter data, and how we estimated each Twitter user’s political ideology, exposure, and receptivity to news articles. Finally, we explain the simulation of platform interventions designed to limit misinformation.

### Selection of top-trending articles

On 31 weekdays between 2019 November 18 and 2020 February 6, we selected the most popular article published within the previous 24 hours from each of five news streams: liberal mainstream news domains, conservative mainstream news domains, liberal low-quality news domains, conservative low-quality news domains, and low-quality news domains without a clear political orientation. We created our two mainstream news streams by collecting the top 100 news sites by US consumption.^h^ and classifying each as either liberal or conservative according to established scores of media partisanship (79). We then constructed the liberal and conservative mainstream news streams by selecting the top ten news websites by consumption in each ideological category (liberal or conservative). For our low-quality news sources, we relied on the list of low-quality news sources from (80) that were still active in November 2019, which we then subsequently classified into three streams with a panel of three undergraduate research assistants (see Supplementary Material, Methods): liberal leaning sources, conservative leaning sources, and those without a clear partisan orientation. For the mainstream news feeds, we determined the most popular article in each news stream using CrowdTangle, a content discovery and social monitoring platform that tracks the popularity of URLs on Facebook pages. For the low-quality news feeds, we determined popularity using RSS feeds.^i^ This transparent article selection process allowed us to source a balanced sample of five daily top-trending news articles from across the ideological spectrum.

To determine the veracity of the articles, we sent each day’s five selected articles to professional fact-checkers during the initial 24 hours after publication. We hired six professional fact checkers from leading national media organizations to assess each article,^j^ and we classified each article with the modal response of the professional fact checkers (“true,” “false/misleading,” or “could not determine”). This process yielded 37 false/misleading articles and 102 true articles, as well as 16 articles that were removed from analysis because the fact-checkers could not agree on.

### Survey data

To determine how likely people were to believe the articles as they encountered them on social media, we sent each day’s five selected articles to a panel of US respondents (13). Each daily survey was completed by a different group of 140–160 American respondents that were recruited by Qualtrics and balanced on age, gender, partisanship, and education. Every respondent evaluated three articles randomly selected from the day’s five selected articles. As a result, each article was assessed by approximately 90 unique respondents who evaluated these articles within 48 hours of its publication, giving us a measure of real-time belief in these stories. Altogether, 5,072 unique respondents evaluated the 139 articles in our dataset, resulting in 13,582 individual article evaluations.

Collecting evaluations of the most popular news articles directly after publication is a key innovation that allowed us to measure near real-time belief in each article as they were spreading on social media (13). Respondents evaluated each article using a variety of criteria, the most relevant of which was a categorical evaluation question—“What is your assessment of the central claim in the article?”—to which respondents could choose from three responses: (i) True (ii) Misleading/False (iii) Could Not Determine (see Supplementary Material, Text for full survey instrument). For the 37 false/misleading articles, we collected 3,394 evaluations from 2,751 unique respondents. For the 102 true articles, we collected 10,024 evaluations from 5,000 unique respondents.

While our selection of articles was not random or representative, our article selection process allowed us to ask respondents to evaluate the news articles that were highly popular and balanced on source ideology and quality. Importantly, the research team was not involved in selecting the individual articles beyond creating the sampling strategy. We chose to instead focus on top-trending articles because social media companies and society at-large are much more interested in studying and intervening in viral misinformation rather than the average item of misinformation that very few individuals see.

Though our surveys did not specifically target social media users, we believe the respondents’ article evaluations likely mimic how those on a social media platform perceive articles. Our opt-in internet survey (administered by Qualtrics) predominately collected responses from habitual internet users. Therefore, while this recruited sample of respondents may not generalize to the overall population, it does well represent the population online. In addition, we did not find that our opt-in survey suffered from a lack of effort among participants. We ran a separate study that measured whether respondents evaluated articles differently when we offered incentives for correct answers, but found no difference between the responses of people who were offered extra incentives and those were not (Figure S8). A figure depicting this can be found in the Supplementary Material, Text.

This study was approved by an independent ethics board at NYU (IRB-FY2019-3511), and informed consent was obtained from all participants prior to their participation.

### Twitter data

Completely separate from the survey data, we also collected Twitter data for each of 139 true and false/misleading articles. We did this by searching for tweets sharing each article’s URL and collecting all tweets and users who shared a URL within one week after publishing. This process yielded a dataset of 139,734 tweets from 92,514 unique users (hereafter, referred to as “tweeters”). Among these, the shares of true article comprised 94,422 tweets from 72,304 unique users, while the shares of false/misleading articles comprised 45,312 tweets from 27,430 unique users. To estimate who may have been exposed to these tweets, we also collected each tweeter’s friend and follower network. This totaled 128,453,928 unique followers and 21,871,687 unique friends of our tweeters (there is overlap between the set of users in the friend and follower lists).

It is important to clarify that survey respondents and Twitter users are completely separate sets of individuals. The only overlap between the survey data and the Twitter data is the news articles themselves.

### Constructing retweet networks

On Twitter, a user can share an article by either (i) directly tweeting an article link, (i) retweeting another user who shared the article link, or (iii) quote tweeting another user’s tweet of the article link with added extra commentary. Retweets and quote tweets are major features on Twitter that allow information to spread beyond the followers of the original tweeter. Consequently, information on Twitter often reaches new parts of the social network, quickly spreading from the initial sharer to their followers and beyond.

To visualize and track the spread of our selected news articles on Twitter, we constructed retweet networks using established methods for time-inferred information diffusion on Twitter (15, 81). Using data from the Twitter API, this method determines the flow of a retweet using the time and friend/follower networks of users. This is necessary because Twitter data does not directly include information about who retweeted which user and instead only includes information about the original tweet, even if it is part of a chain of several retweets.

To build the retweet network, we inferred the path of a retweet or quote tweet by considering the time it was shared and the involved friend–follower networks. If a user (the “retweeter”) follows the original tweeter, we classify it as a direct retweet of the original article share. If the retweeter does not follow the original tweeter, we then check if any of her friends retweeted the same tweet earlier. We assume that the retweet flowed from the friend who most recently shared the same retweet. If the retweeter neither follows the original tweeter nor has any friends who retweeted the tweet earlier, we consider it a direct retweet of the original tweeter. This situation likely reflects instances where users see and retweet content from users they do not follow, a common occurrence on Twitter where users’ feeds also contain viral tweets and tweets liked by followed accounts. (At the time of our study, Twitter’s feed was primarily composed of posts from one’s social graph as opposed to the “for you” feed that is now available.)

### Estimating user ideology

To characterize the ideology of users in our study, we used an established method that infers a user’s ideology from the news, political, and cultural accounts that they follow (42). This method assumes that users are more likely to follow accounts that align with their personal ideology (42, 78). Therefore, using the known ideology of prominent accounts, the method uses correspondence analysis to estimate a user’s ideology, provided that they follow at least one of the prominent accounts with known ideology. Using this method, we compiled a dataset of estimated ideology scores for as many of the users in our dataset as possible.

To determine the ideology of tweeters in our dataset, we cross referenced each tweeter’s unique user ID in our study against the dataset of scored ideologies. If we were unable to directly calculate the ideology of a tweeter because they did not follow any prominent news, cultural, or political accounts, we estimated it using the mean ideology score of their friends.

In our study, we are interested in measuring how many and what kind of users are exposed to news articles on Twitter. Therefore, we needed to estimate the ideology scores of all followers since they sit “downstream” of tweeters in the directed social network of Twitter. To determine the ideology of the followers in our dataset, we again cross referenced each follower’s unique user ID against the dataset of scored ideologies. Followers without available scores in the ideology dataset were estimated differently than tweeters since we lacked their friend network data. We instead assumed that the follower ideologies of a tweeter would follow a normal distribution (42, 78), allowing us to reasonably estimate the missing follower ideologies based on the follower scores we did have.

We used a Bayesian approach to infer the distribution of follower ideologies for a given tweeter. First, we inferred the population-level distribution of user ideologies using all known ideology scores in our dataset, creating a baseline assumption for Twitter user ideologies that could be used as a prior distribution for individual tweeters. Second, we inferred the distribution of follower ideologies for each individual tweeter.

To estimate the baseline distribution of Twitter user ideologies, we randomly sampled 500,000 ideologies without replacement from all scored users in our dataset, including tweeters and their friends and followers. Because ideology scores tend to be normally distributed around 0 (78), we inferred a normal distribution $N(\mu_{\mathrm{pop}},\sigma_{\mathrm{pop}})$ using Bayesian Hamiltonian Monte Carlo. For the priors, we assumed a normal distribution prior (μ=0, σ=2) for the population mean and an exponential distribution prior (λ=2) for our population standard deviation. We ran the Hamiltonian Monte Carlo inference using No-U-Turn sampler across two chains, each with a burn-in of 1,000 iterations and a combined total of 3,000 samples from the posterior.

Using the posterior of our population estimate as our prior distribution, we then inferred the distribution of follower ideology scores for each unique tweeter. For each tweeter, we inferred a skew normal distribution $SN(\mu_{i},\sigma_{i},\alpha_{i})$, allowing for asymmetric distributions. For $\mu_{i}$ (analogous to mean) and $\sigma_{i}$ (analogous to standard deviation), we used the posterior of the population’s $\mu_{\mathrm{pop}}$ and $\sigma_{\mathrm{pop}}$ as the prior, allowing us to assume that, in the absence of strong evidence, a tweeter’s followers tend to look like the population as a whole. For the prior for distribution skew $\alpha_{i}$, we assumed a normal distribution N(0,1), meaning that a user’s followers are most likely to resemble a normal distribution but can skew left or right with equal likelihood. We again ran the Hamiltonian Monte Carlo inference using No-U-Turn sampler, this time across four chains, each with a burn-in of 500 iterations and a combined total of 2,000 samples from the posterior. For the 2,678 tweeters (3.03% of all tweeters) that had no followers with known ideology scores and therefore no data we could use for inference, we used our prior distribution and assumed their followers had ideologies that matched that of the population. Users who did not have any followers with known ideology scores tended to have very few followers (mean follower count <5) and therefore minimally impact the analyses in our study.

### Estimating user exposure and receptivity to news articles

In our study, we sought to estimate the number of Twitter users likely to see and believe various news articles. To do this, we calculated $r_{j}$, the number of users who are exposed and receptive to believing article *j* to be true. The calculation followed this logic:


$$r_{\;j}=\sum_{i}x_{ij}p_{ij}$$

Here, $x_{ij}$ is the count of users within ideological category *i* who were exposed to article *j*, and $p_{ij}$ is the probability that users within ideological category *i* believe article *j* to be true. We derived $x_{ij}$ from the social media data and $p_{ij}$ from the surveys. We will now go in to detail on how these values are calculated.

#### Estimating user exposure $x_{ij}$

To estimate the user exposure metric $x_{ij}$, we needed to determine when and how users encountered particular news articles. We made the simplifying assumption that all followers of a user are potentially exposed to the user’s tweet. We recognize that this is not how exposure occurs in real life, but we made this simplifying assumption because the Twitter API does not provide information on which users saw a tweet. Without detailed knowledge of Twitter’s news feed ranking algorithm, we opted for the most basic dynamic possible. As a result, our measurement of exposure is quantified as *potential exposure*, the maximum number of users who might have seen a tweet. We calculated exposure on a per article basis, considering exposure as a one-time event. This meant that a user could only be “exposed” to an article once, even if multiple accounts that they follow shared the article.^k^

To estimate when users were exposed to each article, we assigned an exposure time to each follower of a user who tweeted the URL for article *j*. We assumed that users are exposed to a tweet with some delay. Thus, for each follower, we calculated their “exposure time” by randomly adding a time delay to the timestamp of the tweet. We drew each follower’s time delay (in hours) at random from a truncated normal distribution (μ=1, σ=2, and minimum limit of 0).^l^ If a user followed multiple tweeters who shared article *j*, we calculated the exposure time for each article tweet and kept the one with the earliest exposure time. In the end, this process allowed us to estimate the number of unique users that were potentially exposed to article *j* and their individual exposure times.

After estimating which Twitter users were exposed to each news article, we then estimated the ideologies among those exposed. In this paper, we use ideology as the predictor of article belief because it is one of the best predictors of belief in misinformation (13, 21–24) and can be reliably estimated from social media data (42). Unfortunately, the other largest predictor from previous literature (13)—familiarity with the political narrative presented in the article—is much harder to infer from Twitter data, but future work could attempt to incorporate information in this regard as well. We inferred the ideologies of exposed users in a two-step process: (i) using the known user ideology scores that we do have, and (ii) drawing the remaining ideologies as samples from that tweeter’s estimated distribution of follower ideologies (see “Estimating user ideology”). For example, if we estimate that tweeter *@ExampleUser* tweeted an article and exposed 1,000 users, and we have known ideology scores for 200 of these users, we would then draw the remaining 800 scores from the estimated ideological distribution of *@ExampleUser*’s followers.

To finish calculating user exposure $x_{ij}$, we needed to bin the ideology scores of exposed users into ideology categories. This step was essential because the surveys determining the rate of belief in article *j* used ideological categories—“Very Liberal,” “Liberal,” “Somewhat Liberal,” “Moderate,” “Somewhat Conservative,” “Conservative,” “Very Conservative”—instead of a continuous numeric scale. External data show that these self-reported ideological categories align closely with ideology inferred from Twitter data (see Supplementary Material, Text; Figures S9, S10). Therefore, we translated each Twitter user’s numeric ideology score into these survey categories by dividing the ideology score space into evenly sized buckets (Table 1).

**Table 1.** Mapping of continuous ideology scores onto ideology categories.

Category	Ideology scores
Very Conservative	x>2.5
Conservative	1.5<x≤2.5
Somewhat Conservative	0.5<x≤1.5
Moderate	−0.5<x≤0.5
Somewhat Liberal	−1.5<x≤−0.5
Liberal	−2.5<x≤−1.5
Very Liberal	x≤−2.5

This categorization schema allowed us to then use our survey data to estimate the proportion of exposed users that was likely to believe particular article *j*.

#### Calculating probability of belief $p_{ij}$

To calculate this probability of belief $p_{ij}$, we used data from the surveys that asked panels of random people to assess the veracity of an article (see “Survey data”). Because respondents provided personal demographic data, we could calculate the proportion of respondents within each ideological category *i* that believed a given article *j* to be true. This yielded a value between 0 (no one the category believes it to be true) and 1 (everyone in the category believes it to be true). Importantly, $p_{ij}$ is derived entirely from the surveys and is independent of how we graded an article’s veracity or ideological slant.

#### Calculating the full estimate of exposed and receptive users $r_{\;j}$

To calculate the full estimate of the number of exposed users who were receptive to believing an article to be true $r_{ij}$, we by multiplied the number of exposed users in an ideology category, $x_{ij}$, by the corresponding probability of belief, $p_{ij}$. For example, we might estimate that a given tweet of article *j* exposed 50 “very liberal” (VL) users, that is $x_{\mathrm{VL},j}=50$. Because we know that 70% of “VL” respondents in the survey believed the article to be true ($p_{\mathrm{VL},j}=0.7$), we estimate that $r_{\mathrm{VL},j}=50\times 0.7=35$ of the “VL” exposed users were receptive to believing the article to be true. Importantly, we describe our measurement as “receptivity” because we cannot causally measure the belief among these social media users. Instead, we can concretely estimate who was both exposed and *likely* to believe the article, based on what we learned from the survey data.

Because our estimation process used random sampling—e.g. drawing exposure time or missing follower ideologies—we recalculated total user exposure $x_{ij}$ and receptive user exposure $r_{j}$ for each scenario, e.g. for each simulated intervention described in “Simulating platform interventions to limit misinformation”.

Our approach balances conservatism and comprehensiveness. On one hand, we only counted users who are likely to affirmatively believe an article, while we did not count users who are unable to discern whether an article is true or false, even though their uncertainty highlights a real challenge in navigating online information. On the other hand, we assume that all followers are eventually exposed, which likely overestimates actual exposure. Therefore, our method provides a conservative upper bound on potential exposure among users receptive to misinformation. Future work can refine the accuracy of our method.

### Simulating platform interventions to limit misinformation

Social media platforms, including Twitter, have implemented measures to limit the spread of misinformation. While individual-level experiments inform us on how certain interventions may reduce the likelihood that an individual shares a piece of misinformation, we still do not have an understanding of how these interventions work at scale, particularly in preventing misinformation exposure among the users most likely to believe it.

We used our dataset to conduct simulations that test how misinformation exposure among receptive users is affected by (i) the speed of fact-checking and (ii) the method of intervention. Since external professional fact-checkers must first label an article as false/misleading before an intervention can be deployed, we focused our simulations on the articles in our dataset that were rated false/misleading by fact-checkers. Since interventions can be slowed down by the fact-checking process and other logistics, our simulations considered the number of hours of delay, $t_{\mathrm{int}}$, it takes to deploy an intervention after the first share of a targeted article’s URL. We varied the intervention time $t_{\mathrm{int}}$ to see how the intervention speed impacts its effectiveness in decreasing misinformation exposure.

We simulated two methods that are commonly used to try to reduce the sharing of misinformation: *fact-check labeling* and *sharing friction*. Fact-check labeling involves attaching warning labels to tweets sharing misinformation. We assumed that fact-check labeling makes a user 25% less likely to retweet (17) and 17% less likely to be receptive to (36) a flagged tweet after time $t_{\mathrm{int}}$. Sharing friction adds extra steps (e.g. more clicks) to the retweet process in the hope that users will reconsider sharing a questionable content. We assumed that sharing friction makes a user 75% less likely to retweet (17) a flagged tweet after time $t_{\mathrm{int}}$. In a simulation of an intervention on a particular false article, we took our set of real article tweets and removed a retweet after time point $t_{\mathrm{int}}$ with probability 0.25 (fact-check labeling) or 0.75 (sharing friction), thereby simulating the prevention of a retweet due to the intervention. When a retweet was removed, we removed all subsequent retweets of that specific tweet, since users would be unable to see and retweet a tweet that did not occur. Additionally, in the case of fact-check labeling, we decreased the survey-based belief rates by 17% when estimating which exposed users were receptive to believing the misinformation to be true (see “Estimating user exposure and receptivity to news articles”).

We also simulated *visibility reduction*, where tweets sharing questionable content are “less visible in other user’s timelines,” as stated in Twitter’s earlier terms of service. Therefore, we simulated visibility reduction by making it 25% (light visibility reduction) or 75% (heavy visibility reduction) less likely that a user sees tweets sharing flagged articles after time $t_{\mathrm{int}}$. Unlike the simulations of sharing-focused interventions, these percentages were not based on the literature but instead represented comparable values that represent varying degrees of intervention aggressiveness. We simulated this intervention during the exposure calculation for a given false/misleading news article: taking the list of users that we estimated were exposed to the article, we would probabilistically remove users exposed after time $t_{\mathrm{int}}$, prior to dropping duplicate exposures of the same user (see “Estimating user exposure and receptivity to news articles”). This approach allowed us to realistically capture instances where a user may miss the first exposure chance due to the intervention but later see it if they are following multiple accounts sharing the article.

## Supplementary Material

pgae396_Supplementary_Data

## Data Availability

All article and Twitter data, exposure estimates, and simulation results can be accessed at Zenodo at 10.5281/zenodo.13777170. All analysis and simulation code used in this paper is available on Zenodo at 10.5281/zenodo.13777145 for a permanent archived version. The most up-to-date version, if any changes occur post-publication, will remain accessible on GitHub at https://github.com/christokita/news-belief-at-scale.

## Notes

a Misinformation fueled riots at the US Capitol On 2020 January 6 (82, 83) and at the Brazilian Capitol on 2023 January 8 (84, 85), threatening the transfer of power in both countries following recent elections.

b Waves of misinformation about COVID-19 have increased distrust of public health officials, weakened responses to the pandemic, and increased skepticism of the COVID-19 vaccine (86, 87).

c Tables S1–S5 list the articles used in the study by veracity, source type/learn, topic, and article slant; the method by which the articles were selected can be found in the Material and methods section “Selection of top-trending articles”.

d Mainstream left leaning publications, mainstream right leaning publications, low-quality left leaning publications, low-quality right leaning publications, and low quality publications without an apparent ideological lean across articles.

e Sixteen articles were removed from the study because they either received a rating of “could not determine” or because there was no modal rating among the fact-checkers. See the Materials and methods section for details.

f See https://docs.anthropic.com/en/docs/content-moderation

g See https://platform.openai.com/docs/guides/moderation

h These were identified by Microsoft Research’s Project Ratio between 2016 and 2019: https://www.microsoft.com/en-us/research/project/project-ratio/.

i We used RSS feeds for the low-quality sources because most low-quality sources were not tracked by CrowdTangle, as the publisher pages had been removed from the platform; for more on CrowdTangle see https://www.crowdtangle.com/.

j These professional fact-checkers were recruited from a diverse group of reputable publications (none of the publications that we ask individuals to fact-check to ensure no conflicts of interest) and paid $10.00 per article.

k Future work could relax this simplifying assumption to account for the likely scenario that users may see an article multiple times if more than one of the people they follow shares it.

l While we explored this simplified assumption, future work could explore different assumptions for how exposure occurs over time.
