# Supplementary material for: Measuring receptivity to misinformation at scale on a social media platform
Source: PNAS Nexus. 2024 Sep 10;3(10):pgae396. doi: 10.1093/pnasnexus/pgae396 (PMC11460357; doi:10.1093/pnasnexus/pgae396)
Supplement: pgae396_Supplementary_Data [file pgae396_supplementary_data.pdf]

Supplementary Material for:
Measuring receptivity to misinformation at scale on a social media platform

Christopher K. Tokita[1,*], Kevin Aslett[2,4], William P. Godel[2], Zeve Sanderson[2], Joshua A. Tucker[2,3], Jonathan Nagler[2,3], Nathaniel Persily[6], and Richard Bonneau[2, 5]

[1]Department of Ecology and Evolutionary Biology, Princeton University, Princeton, NJ, USA
[2]Center for Social Media and Politics, New York University, New York, NY, USA
[3]Department of Politics, New York University, New York, NY, USA
[4]School of Politics, Security, and International Affairs, University of Central Florida, Orlando, FL, USA
[5]Prescient Design, a Genentech accelerator, New York, NY, USA
[6]Stanford University Law School, Stanford University, Palo Alto, CA, USA
[*]email: christopher.tokita@gmail.com

1 Supplementary Text

1.1 Tweeting

We find that Twitter users with more extreme ideologies were more likely to share news articles than users with moderate ideologies (Figure S2A). Additionally, most article shares were in the form of retweets rather than original shares of the article URL. Among retweets, most were direct retweets of the original tweeter rather than indirect retweets from a friend-of-a-friend (Figure S11). This pattern suggests that information on Twitter does not typically spread far beyond one degree of separation from the original sharer. This sharing pattern matches the expected behavior of information cascades on Twitter (1, 2), which exhibits a hub-and-spoke (i.e., scale-free) network structure. We explore these cascades in the next section.

1.2 Diffusion of news articles through social networks

By constructing retweet networks, we find that the news diffusion on Twitter is both structurally and politically skewed (Figure S2D). A few users, often news outlets or prominent figures with large followings, garner most of the retweets (Figure S12). This reflects the scale-free nature of Twitter's social networks (1, 3), where a few nodes (users) have a disproportionate number of connections and thereby act as major hubs of information dissemination.

Additionally, we find that retweet networks are relatively politically homogeneous. Articles tend to be tweeted within ideologically similar sets of users. Interestingly, the social networks that false/misleading news articles pass through tend to be less ideologically diverse than those sharing true news ($BF_{10} \gg 100$, $p(\mu_1 \neq \mu_2) = 1$; $t(52.412) = -2.11$, $p = 0.0396$) (Figure S2C). This finding may reflect the homophily observed in social networks on Twitter (Figure S13A): left-leaning users tend to have left-leaning followers, right-leaning users tend to have right-leaning followers, and moderate users tend to have the most ideological diverse set of followers (Figure S13B). Thus, because ideological extreme individuals are more likely to share news articles from fringe news sources—the main source of false/misleading news—and are more likely to have politically similar followers, false/misleading news articles tend to be introduced to politically uniform social networks.

We also find some political asymmetry in the social network structure on Twitter. While moderate users did tend to have the most ideological diverse set of followers, center-right users tended to have more diverse audiences than their center-left counterparts (Figure S13B). As a result, we find that center-right users generated a disproportionate amount of exposure across ideological lines (Figure S14), that is, articles they shared often exposed both conservative and liberal users.

Draft

1.3 User ideology as a predictor of belief in news articles

Previous work from (4) demonstrated that user ideology is a significant predictor of belief in news article content, particularly misinformation. Their study used the same survey dataset as ours, where participants rated top-trending news articles as true or false/misleading. Among the demographic information collected from survey respondents, age, cognitive reflection, and ideological congruence between a user and an article's content were statistically significant predictors of believing an article to be true (Figure S1A). Participants were, on average, 16% more likely to believe a piece of misinformation if its content aligned with their personal ideological slant. Conservative participants were far more likely to believe a piece of misinformation to be true if the article had a right-leaning slant, and liberal participants were similarly more likely to believe a piece of misinformation to be true if the article had a left-leaning slant (S1B). This finding, along with parallel results in the literature (5–8), informed our decision to focus on user ideology as the predictor of belief in our study. Among the demographic characteristics measured in (4), user ideology had the strongest effect on user belief and was also the only statistically significant characteristic that could be readily measured in our Twitter data.

However, in our study, we did not use these top-level estimates of the effect of ideology on belief. Instead, we calculated the likelihood of belief based on ideology for each article independently using the survey data, regardless of the article's slant. For a given article, we calculated the percentage of survey participants in each ideological category who believed the article to be true. As a result, each article has an estimated belief rate for each of the seven ideological categories that a user could self-identify. This approach allows for the possibility that two articles with the same ideological slant and veracity could have very different belief rates across ideological categories.

1.4 Users' Twitter ideologies align with their self-reported ideologies

To verify the accuracy of user ideologies inferred from Twitter data, we analyzed an outside dataset from YouGov, a major public opinion research firm. This dataset came from a survey conducted from January 23rd, 2020 and February 18th, 2020, in which 941 U.S. respondents consented to share their political attitudes and Twitter account information. Using the respondents' Twitter information, we then inferred the survey-takers' ideologies using the established method (9, 10) that we used throughout our study. We found general alignment between a respondent's self-reported ideology and the ideology inferred from solely their Twitter account. This confirms that we can accurately infer a user's actual ideology from their Twitter data. Similarly, it shows that by mapping inferred ideology scores onto the categories used in our study's survey, we can reliably infer individual receptivity to news articles based on survey data.

A key difference in the YouGov dataset was its five-category scale ideology scale: "Very Liberal", "Liberal", "Moderate", "Conservative", or "Very Conservative". This scale contrasts our study's seven-category scale, which also included "Somewhat Liberal" and "Somewhat Conservative" options. Due to YouGov's coarser ideology scale, we can reasonably assume that respondents predisposed to select a "Somewhat" category had to instead select "Moderate" or a more extreme category (i.e., "Liberal" or "Conservative").

To address this lack of "Somewhat Liberal" and "Somewhat Conservative" categories, we used two methods for calculating the ideological mean for the missing "Somewhat" categories. First, we calculated a synthetic mean for "Somewhat" categories by averaging the ideologies of users in the adjacent categories (i.e., "Moderate" and "Liberal", or "Moderate and "Conservative"). Alternatively, we created synthetic user groups: one-third of "Liberal" and "Moderate" users were randomly reassigned to "Somewhat Liberal", and one-third of "Conservative" and "Moderate" users were randomly reassigned to "Somewhat Conservative". We then included these synthetic groups when calculating the mean of all ideological categories.

Regardless of the approach, we find consistent results: self-reported ideologies closely matched those inferred from Twitter data (Figure S9). Using Twitter data, we were able to accurately classify most users on the correct end of the left-right political spectrum: 83.4% of users who self-identified as left-leaning were correctly classified as some degree of "Liberal" and 79.6% of right-leaning users were correctly classified as some degree of "Conservative". Broad ideological mix ups were infrequent, with only 4.9% of left-leaning users labeled as some degree of "Conservative" and 10.4% of right-leaning users labeled as some degree of "Liberal". However, when inferring ideologies from Twitter data, there is a tendency to over categorize left-leaning users as simply "Liberal"—as opposed to "Very Liberal" or "Somewhat Liberal"—and likewise a tendency to over categorize right-leaning users as "Conservative" (Figure S10A).

Fortunately, because we can accurately place users broadly on the left-right political spectrum, our estimates of user receptivity to news articles remain reasonably accurate. When examining how participants in our survey rated the veracity of top-trending news articles, we see that adjacent ideological categories tended to believe articles at a similar rates (Figure S10B). For example, users who self-identify as "Slightly Liberal" and "Moderate" tended to believe articles at similar rates, as did users who identified as "Liberal" and "Somewhat Liberal" or users who identified as "Somewhat Conservative" and "Conservative". However, we observed greater differences in belief rates across ideological categories for False/Misleading news compared to True news.

2 Supplementary Methods

2.1 Coding the partisan slant of news sources and articles

To determine the partisan lean of low-quality news sources, we used a panel of three undergraduate research assistants who served as independent coders. The coders were tasked with classifying each low-quality news source as either "liberal", "conservative", or "unclear". They each independently made their decision using information found in a news source's headlines, article content, website "about" page, and, if applicable, known partisan affiliations. A source was only labeled as "unclear" if fewer than 50% of a news source's content appeared to have partisan content. When coders did not unanimously agree on the partisan slant of a news source, a fourth coder was brought in and the majority rating was used. Coders agreed unanimously on 75% of news sources. In the end, six low-quality sources were classified as "liberal", fifty were classified as "conservative", and forty three were classified as "unclear".

For determining the partisan slant of individual articles, we used a panel of four undergraduate research assistants, again serving as independent coders. For a given article, we tasked each coder with viewing only the article's title and body text, and then rating the article as "liberal", "conservative", "neutral", or "unclear". We used the modal rating of the four coders as each article's partisan slant. In the event that there was a tie, a graduate student coder served as a tiebreaker.

3 Supplementary figures

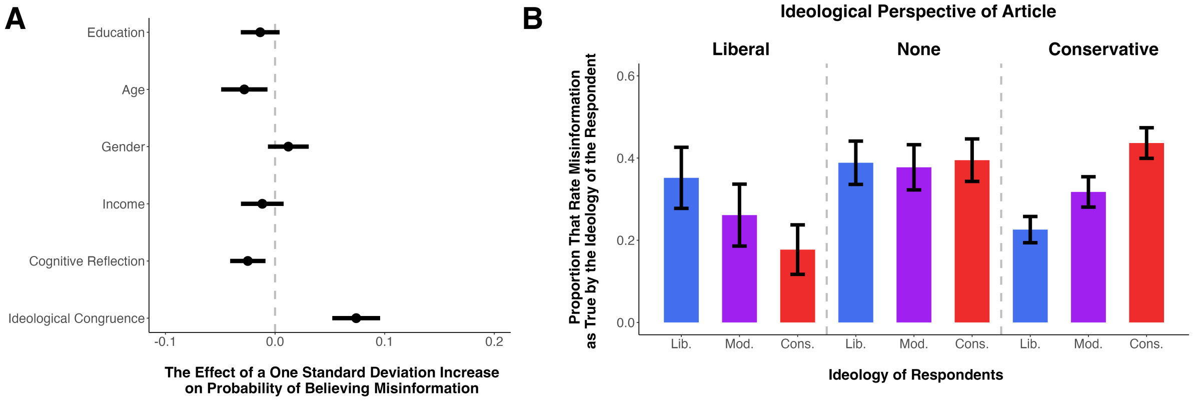

Figure S1: User ideology and ideological congruence with article content are a significant predictor of individual belief in misinformation. Figures are adapted from (4) with permission. (A) Individual-level demographic characteristics associated with believing misinformation to be true. Points represent the estimated effect size and 95% confidence interval of a linear regression model. (B) Estimated proportion of users who believed a piece of misinformation to be true, broken out by participant ideology and article slant. Bars represent the 95% confidence interval.

Draft

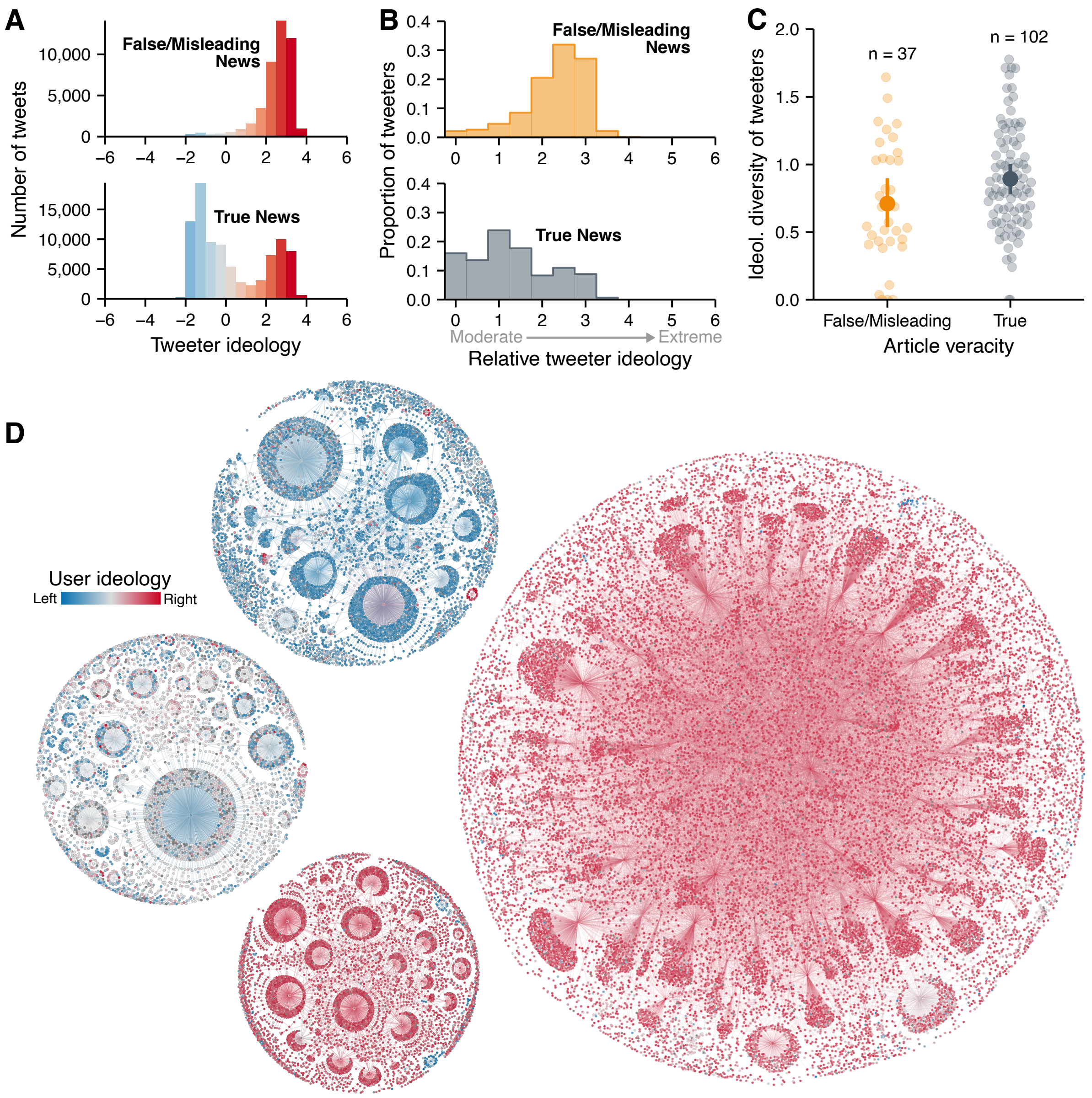

Figure S2: The pattern news articles sharing and diffusion on Twitter. (A) Histogram showing the distribution of ideologies among sharers of true and false news. Negative (blue) values denote left-leaning ideology, while positive (red) values denote right-leaning ideology. (B) Ideological extremity among sharers of true and false news. Lower values indicate users with slightly left-leaning or right-leaning ideologies, while higher values indicate users with more extreme left-leaning or right-leaning ideologies. (C) The ideological diversity among tweeters of true and false news articles, measured by the standard deviation of ideology for all tweeters of a specific article. Small points represent the ideological diversity of individual articles, while the large points represent the mean ideological diversity (±99% credible interval) across all true or false news articles. (D) Example retweet networks for individual articles in our data set. Each node is a tweeter of the article, colored by their ideology, with arrows depicting the direction of retweets.

Draft

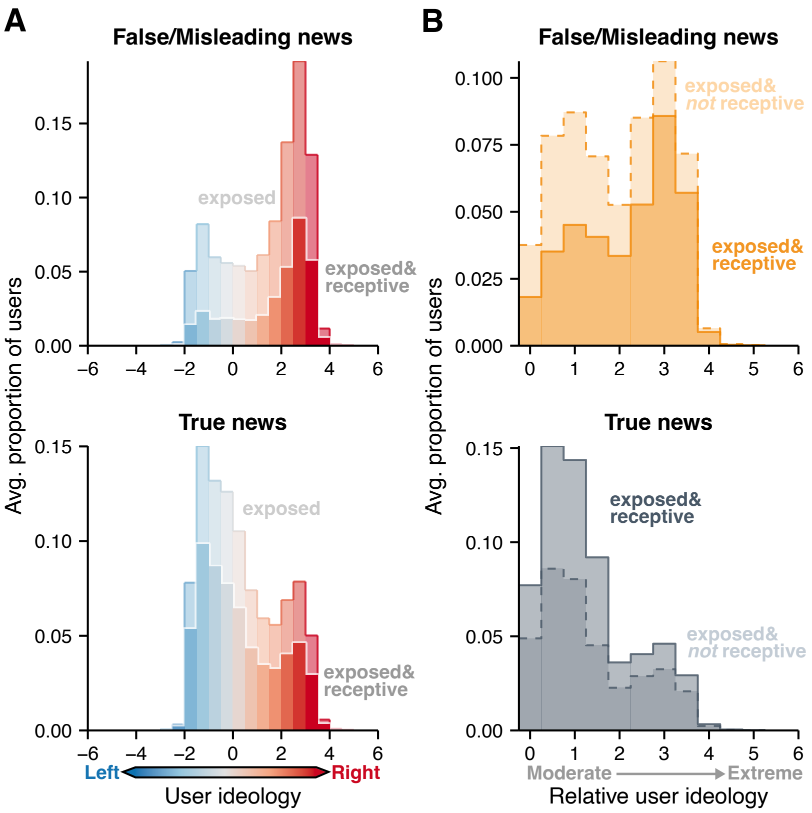

Figure S3: Average distribution of user ideology among those exposed and receptive to top-trending news articles on Twitter, normalized by article virality. To control for differences in the total number of users exposed to each article, we first calculated the proportion of exposed users within each ideological bin for every article. We then averaged these proportions across all articles of a given type (true or false/misleading) to obtain the average ideological distribution of exposed users. (A) The average distribution of user ideology among those potentially exposed to true and false/misleading news articles. Negative (blue) values indicate left-leaning ideology, while positive values (red) indicate right-leaning ideology. (B) The average distribution of ideological extremity among users potentially exposed to news articles. Low values indicate users with slightly left-leaning or right-leaning ideology, while large values indicate extreme left-leaning or right-leaning ideology.

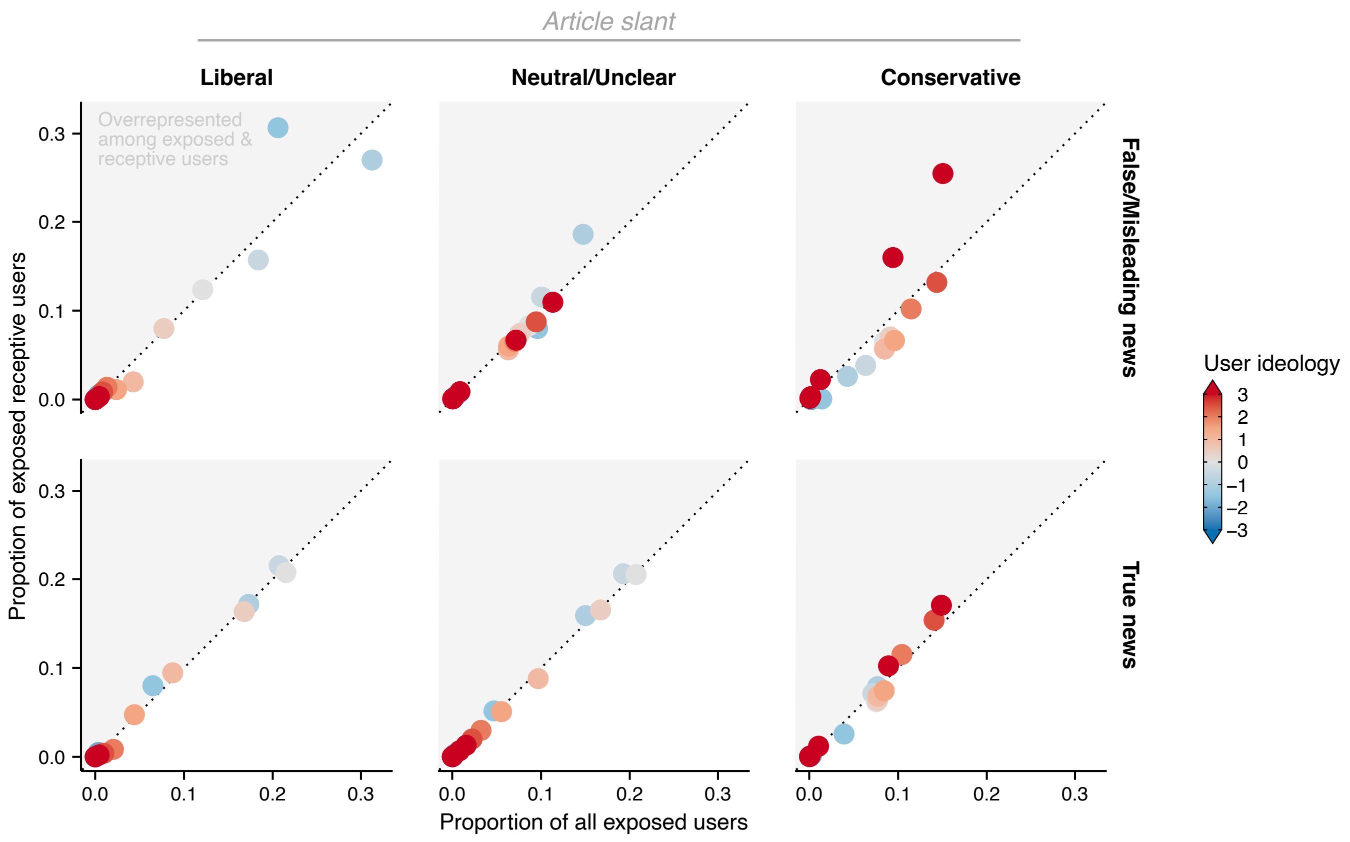

Figure S4: The relative abundance of a given ideology among all exposed users vs. all exposed receptive users, broken out by article content. For user ideology, negative (blue) values indicate left-leaning ideology and positive values (red) indicate right-leaning ideology. The dashed line represents a 1:1 ratio, indicating that users of that ideology represented a proportion of exposed receptive users that is expected given the proportion they made up of all exposed users. Points above the line indicate that users of that ideology make up a disproportionate share of receptive users among those exposed to an article.

Draft

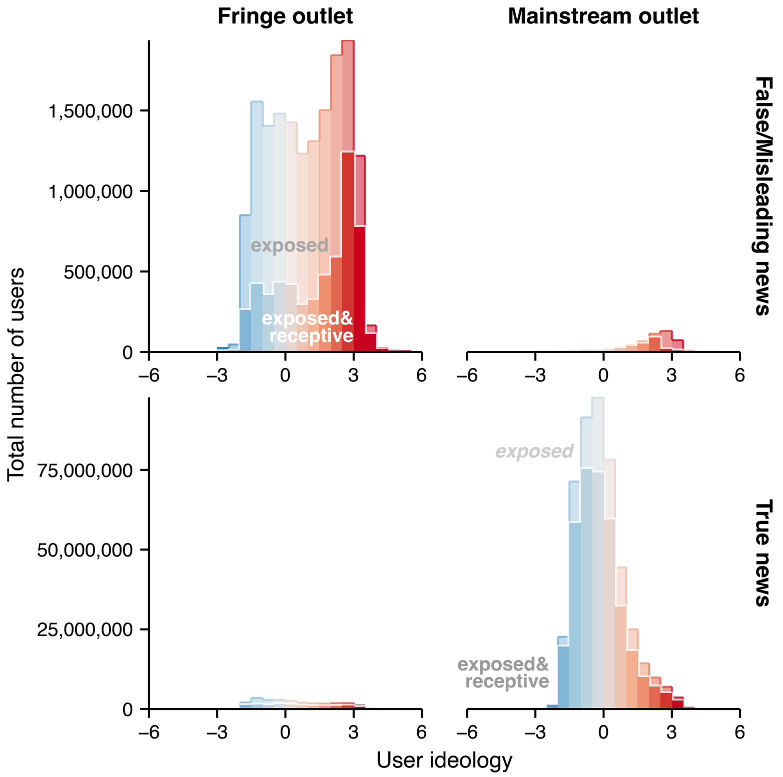

Figure S5: Total number of users exposed and receptive to believing articles to be true, broken out by news source type. Negative (blue) values represent users with left-leaning ideologies, while positive (red) values indicate right-leaning ideologies.

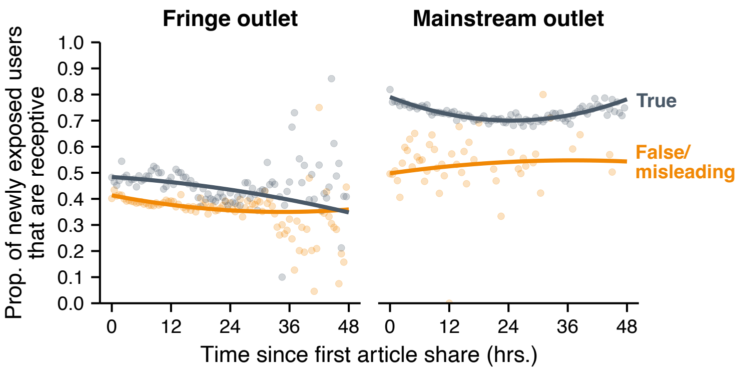

Figure S6: The temporal dynamics of receptivity among newly exposed users over time. This plot shows the proportion of newly exposed users who are receptive to believing an article to be true, broken out by article veracity and news source type. The lines are the best-fit Bayesian regression models applied to all raw Tweet data. For ease of visualization, the points show the binned mean of tweets aggregated within 30-minute intervals.

Draft

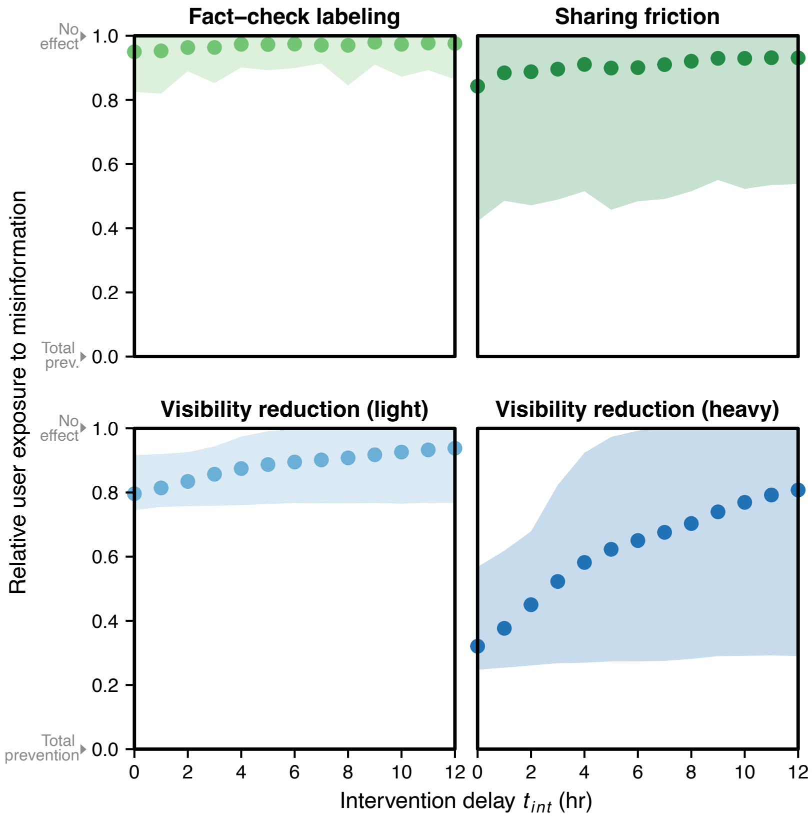

Figure S7: Simulating how intervention method and timing reduce total user exposure in misinformation. Intervention delay t_{int} is the number of hours between an article's publication and the initiation of an intervention. Fact-check labeling decreases the probability of retweets by 25%, while sharing friction—adding extra steps to the retweet process for tweets sharing flagged material—decreases the probability of retweets by 75%. Visibility reduction decreases the chance of a tweet with a flagged news article appearing in other users' feed by 25% (light) or 75% (heavy). The points on the plot show the average results from data-driven simulations across all fake news articles in our dataset, with each ribbon encompassing 90% of all simulation outcomes. Each article was simulated 10 times for a given intervention type and delay.

Draft

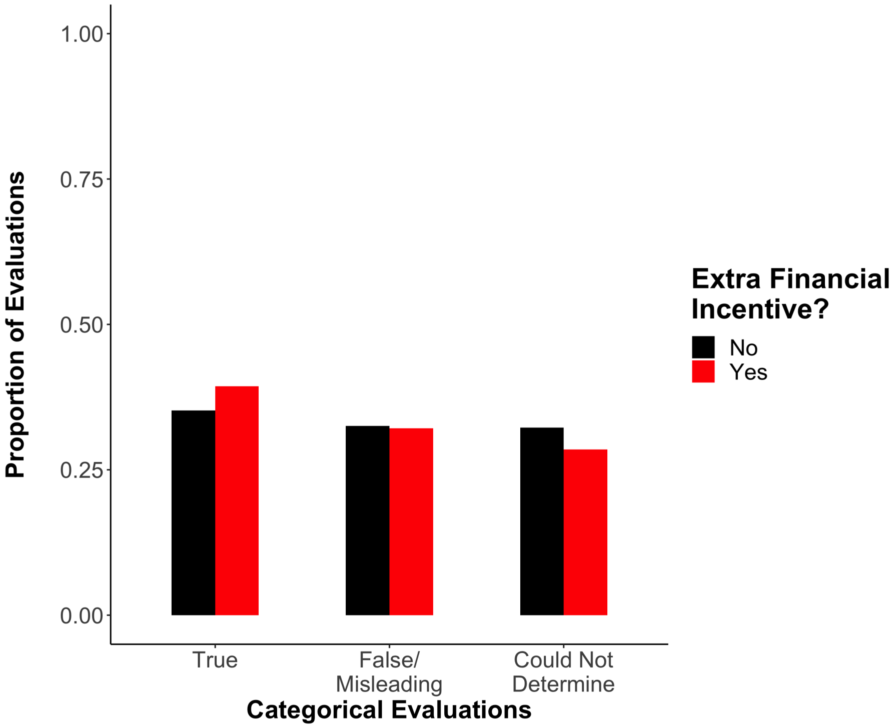

Figure S8: Financial incentives do not significantly affect evaluations from survey respondents. This plot compares the proportion of evaluations for false/misleading news articles between respondents who were offered extra financial incentives and those who were not.

Draft

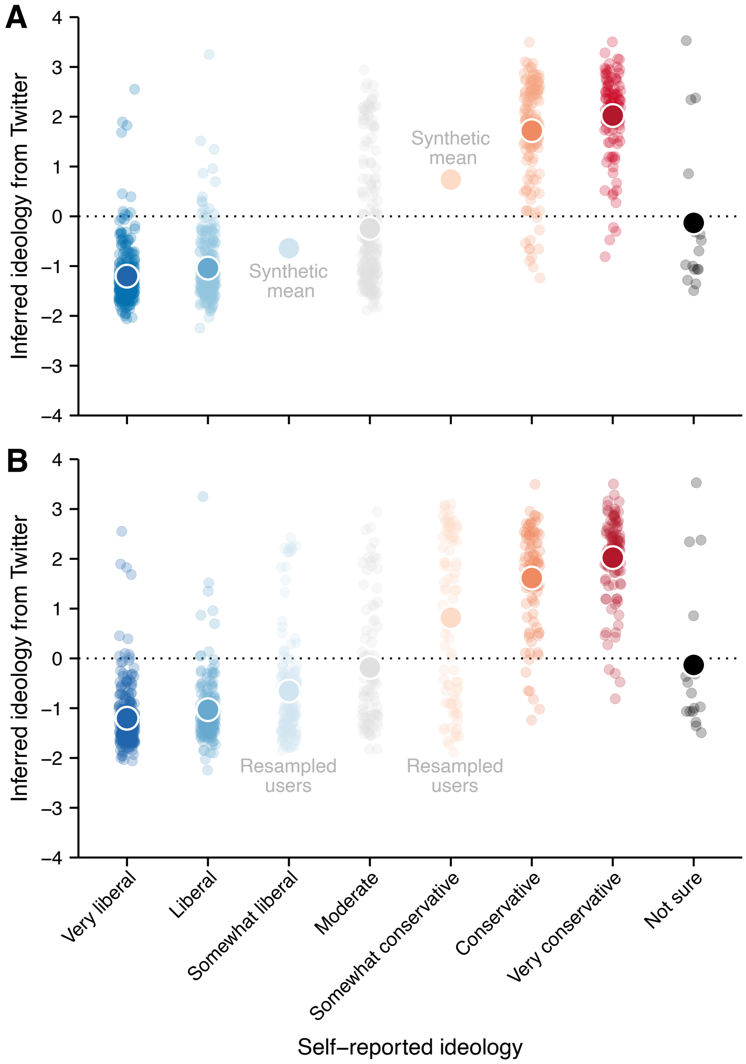

Figure S9: User ideology inferred from Twitter data aligns with self-reported ideology. To verify the accuracy of user ideology inferred from Twitter, we used self-reported ideology data from a YouGov survey in which respondents ($n = 941$) provided their own ideology and their Twitter account information. We then inferred their Twitter ideology using this information. The survey did not include "Somewhat Liberal" and "Somewhat Conservative" categories, so we calculate their ideological mean using two approaches: (A) calculating a synthetic mean from all users in the adjacent categories (e.g., combining 'Moderate' and 'Liberal' for 'Somewhat Liberal'), and (B) sampling and reassigning some users from the adjacent categories. See the Supplemental Text section for more details.

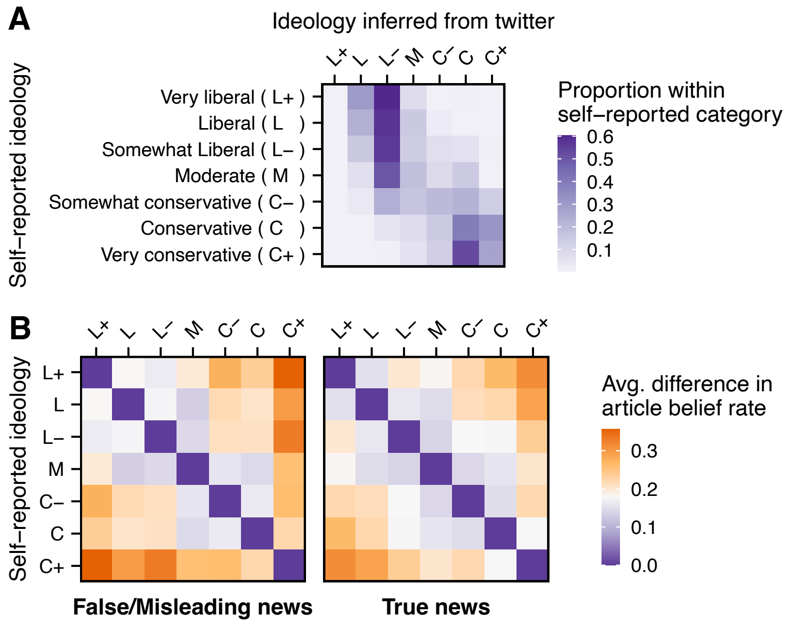

Figure S10: User ideologies inferred from Twitter generally align accurately with users' positions on the left-right spectrum, aiding in precise estimates of user receptivity to news articles. (A) Confusion matrix comparing self-reported ideology and Twitter-inferred ideology for participants in the YouGov survey (n = 941). The "Somewhat Liberal" and "Somewhat Conservative" are resampled data, as in Figure S9B. This matrix helps to assess the accuracy of inferring a user's ideology based on their Twitter activity. (B) Average difference in belief rates across users with different ideologies (n = 5,072) in the surveys where participants evaluated the veracity of top-trending news articles in our study. Lower values suggest that users across the two ideological groups tended to believe the articles at similar rates.

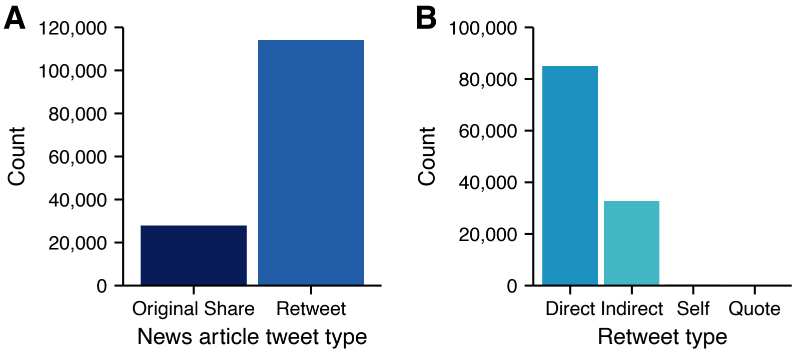

Figure S11: (A) Breakdown of URL share types on Twitter in our dataset. (B) Detailed breakdown of the retweet types in our dataset. We categories retweets into four distinct types: direct retweets occur when a user retweets the original sharer of a link; indirect retweets occur when a user retweets someone who had already retweeted the original sharer, essentially forming a retweet chain; self-retweets happen when a user retweets their own tweet; and quote tweets are a unique form of retweet where a user retweets another user's post while adding their own commentary or text

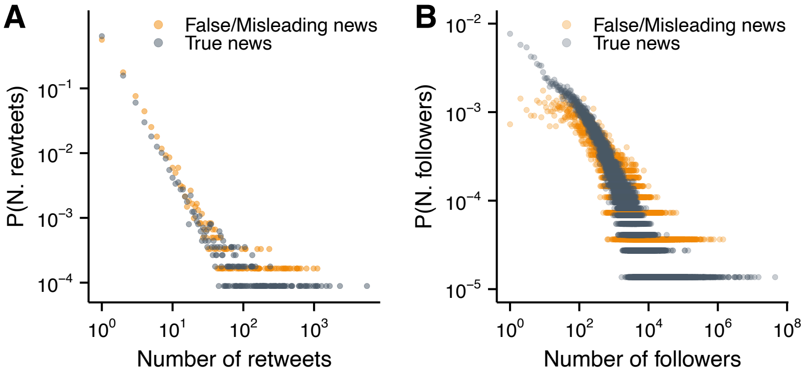

Figure S12: (A) Distribution of retweet frequencies of news articles. This plot presents the frequency distribution of tweets having N retweets of a given article, broken out by true and false/misleading news articles. (B) Degree distribution of users who shared true and false/misleading news articles. This plot depicts the frequency distribution of tweeters having K followers, comparing those who shared false/misleading news articles with those who shared true news articles. This provides insight into the follower base sizes of users disseminating information on Twitter

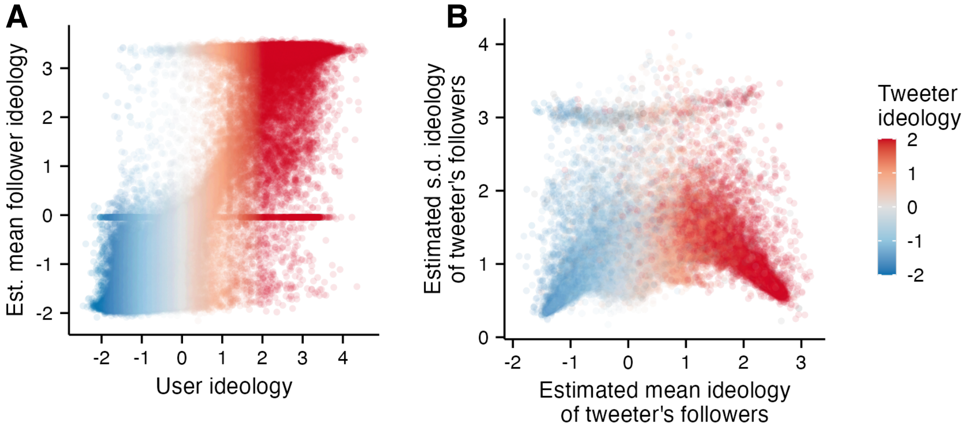

Figure S13: The structure of the follower network on Twitter is ideologically sorted, but center-right users have the most ideologically diverse followers. Negative (blue) values represent left-leaning ideology, while positive (red) values represent right-leaning ideology. (A) Correlation between a tweeter's ideology and the estimated mean follower ideology. The positive relationship would suggest homophily along political lines, where a user's have followers with similar ideologies. (B) The diversity of follower ideologies, as demonstrated by the estimated mean and standard deviation of follower ideology for all unique Tweeters in our dataset. Higher values on the y-axis indicate that the user has more ideologically diverse followers. More extreme values on the x-axis suggest users whose followers predominantly lean strongly towards a particular political ideology.

Draft

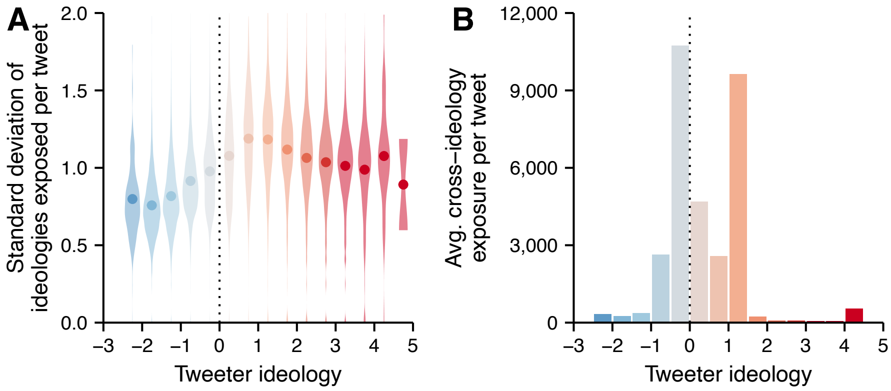

Figure S14: Center-right users exposed the most ideologically diverse audience on Twitter and created a disproportionate amount of cross-ideology exposure to news articles. Negative (blue) values represent left-leaning ideology, while positive (red) values represent right-leaning ideology. (A) The standard deviation of user ideologies exposed to tweets, broken out by tweeter ideology. White points represent the average standard deviation. (B) Average number of cross-ideology exposures per tweet, broken out by tweeter ideology. Cross-ideology exposure is defined as instances where a user on one side of the ideological spectrum (e.g., right-leaning with an ideology score ¿0) exposed a user on the opposite side (e.g., left-leaning with an ideology score ¡0) to a news article.

3.1 Supplementary Tables

Table 1: Headlines for Articles Chosen from the Low Quality Liberal News Stream

	Date	Headline	Modal Fact Checker Rating	Topic	Lean of Article
1	11/18/19	Doctor Tells CNN Trump's Walter Reed Medical Visit Was Fishy	False/Misleading	Political/Economy	Liberal
2	11/19/19	Rudy Giuliani has completely berserk meltdown as Feds close in on him	No Mode	Political/Economy	Liberal
3	11/20/19	Lt. Col. Vindman: 'This Is America...Here, Right Matters'	True	Political/Economy	Liberal
4	11/21/19	Sondland's testimony directly implicates Trump, Pence and Pompeo in Ukraine quid pro quo plot	True	Political/Economy	Liberal
5	12/2/19	Senator Announces Impeach Vote Hint That Has Trump Fuming	No Mode	Political/Economy	Liberal
6	12/3/19	The sealed "Indictment A" that Donald Trump needs to worry about more than ever	False/Misleading	Political/Economy	Liberal
7	12/4/19	Devin Nunes Shamelessly Lies When Hannity Asks About Lev Parnas	False/Misleading	Political/Economy	Liberal
8	12/5/19	Trump caught by reporters patting himself on back for insulting Justin Trudeau	True	Political/Economy	Liberal
9	12/9/19	Ex-Intel Slam Trump For Sucking Up To Saudis After Navy Shooting	True	Political/Economy	Liberal
10	12/10/19	Nancy Pelosi knows something we don't	False/Misleading	Political/Economy	Neutral
11	12/11/19	Tucker Carlson's White Power Hour Guest: AOC's District Is The 'Least American'	True	Political/Economy	Liberal
12	12/12/19	Bush's ethics chief: Trumps are an "organized crime family," we need to "go after all of them"	True	Political/Economy	Liberal
13	12/16/19	Trump Attacks Congresswoman For Not Having His Back	True	Political/Economy	Liberal
14	12/17/19	Donald Trump caught retweeting bizarre fake account	No Mode	Political/Economy	Liberal
15	12/18/19	No Punches Pulled In Climate-Themed Campaign Ad	True	Political/Economy	Liberal

16	12/19/19	Shaken Trump vows Democrats will see backlash at “the box office” after impeachment verdict	True	Political/Economy	Liberal
17	1/6/20	Schiff Hammers President & GOP Over Impeachment Trial Obstruction	True	Political/Economy	Liberal
18	1/7/20	Everything is falling apart for Donald Trump in real time	True	Political/Economy	Liberal
19	1/8/20	Trump bewilders nation by tweeting “all is well” and “so far so good” after Iran’s missile strike	True	Political/Economy	Liberal
20	1/9/20	John Bolton Will Testify If Subpoenaed, So Why Aren’t House Dems Doing That?	No Mode	Political/Economy	Liberal
21	1/13/20	New Trump Approval Poll Released Confirms Massive 2020 Blue Wave	False/Misleading	Political/Economy	Liberal
22	1/14/20	Donald Trump’s GOP Senate allies have just been backed into a no-win corner	No Mode	Political/Economy	Liberal
23	1/15/20	Newly released texts from Giuliani collaborator appear to show them stalking Amb. Yovanovich	True	Political/Economy	Liberal
24	1/21/20	Even C-SPAN Is Cut Off From Covering Senate Impeachment Trial	True	Political/Economy	Liberal
25	1/22/20	Schiff Opening Impeachment Trial Statement To Go Down In History	True	Political/Economy	Liberal
26	1/23/20	Donald Trump just screwed up and blew a gaping hole in his own impeachment trial strategy	No Mode	Political/Economy	Liberal
27	1/27/20	Damning potential John Bolton Ukraine impeachment testimony revealed in early leak of book draft	True	Political/Economy	Liberal
28	1/28/20	Joni Ernst Gives Away The Ballgame On Joe Biden	No Mode	Political/Economy	Liberal
29	2/4/20	Donald Trump’s sham acquittal is already blowing up in Senate Republicans’ faces	No Mode	Political/Economy	Liberal
30	2/5/20	Susan Collins Betrays The Country With Vote To Acquit	True	Political/Economy	Liberal
31	2/6/20	Jennifer Granholm Catches Rick Santorum Shamelessly Lying About Pre-Existing Conditions	False/Misleading	Political/Economy	Liberal

Table 2: Headlines for Articles Chosen from the Low Quality Conservative News Stream

	Date	Headline	Modal Fact Checker Rating	Topic	Lean of Article
1	11/18/19	KANYE WEST AND HIS SUNDAY SERVICE SHOW PERFORM WITH ILLUMINATI ALL-SEEING EYE OF HORUS STAGE SET AT LAKEWOOD CHURCH TO SOLD OUT CROWD	False/Misleading	Human Interest	Unclear
2	11/19/19	Schiff Named in WH Official's Defamation Lawsuit, Leaked Lies To Politico To Push Impeachment	False/Misleading	Political/Economy	Conservative
3	11/20/19	No Shots Fired! Citizen with a Gun Ends Gunman's Attack at Oklahoma Walmart	False/Misleading	Political/Economy	Conservative
4	11/21/19	Indictment Against Head Of Burisma Reveals 'Hunter Biden Was Receiving Payments From Money Raised Through Criminal Means, Siphoned, Laundered From Ukraine'	False/Misleading	Political/Economy	Conservative
5	12/2/19	Montana Gov. Bullock Drops Out Of 2020 Presidential Race	True	Political/Economy	Neutral
6	12/3/19	Donald Trump SLAMS Corbyn's NHS lies 'We want nothing to do with it!'	False/Misleading	Political/Economy	Neutral
7	12/4/19	In 2018, 86% of Those Arrested for Violent Crime in Los Angeles Were Non-White (5% Were White): the City Is 28% White	False/Misleading	Political/Economy	Conservative
8	12/5/19	DING! DING! DING! First Muslim woman elected to Pennsylvania House of Representatives has been ARRESTED for stealing $500,000 from a charity	True	Political/Economy	Conservative
9	12/9/19	NEVER TRUMPER RICK WILSON SUGGESTS PUTTING ANTI-VAXXERS IN "RE-EDUCATION CAMPS"	True	Political/Economy	Conservative
10	12/10/19	Breaking: Ukrainian Official Reveals Six Criminal Cases Opened In Ukraine Involving The Bidens	False/Misleading	Political/Economy	Conservative

Draft

11	12/11/19	Ukraine Advisor Disputes Key Point In Impeachment Testimony — Is This Bad News For Democrats?	False/Misleading	Political/Economy	Conservative
12	12/12/19	NYC's De Blasio Deports Thousands of Homeless Families Across America	False/Misleading	Political/Economy	Conservative
13	12/16/19	Trans Activists Target Olympic Cyclist Inga Thompson For Saying Women Shouldn't Have To Compete With Biological Men	False/Misleading	Human Interest	Conservative
14	12/17/19	Back Home In Pelosi's San Francisco: Homeless Drug Addicts Are Now Taking Dumps In The Supermarket Aisles	False/Misleading	Political/Economy	Conservative
15	12/18/19	Video of the Day: Dem Rep Raskin thanks Congressman helping form rules for sham impeachment of Trump who was impeached for bribery	False/Misleading	Political/Economy	Conservative
16	12/19/19	These Democrats Voted AGAINST Impeaching Trump	False/Misleading	Political/Economy	Conservative
17	1/6/20	NEARLY 200 PEOPLE ARRESTED ACROSS AUSTRALIA FOR DELIBERATELY STARTING BUSHFIRES	False/Misleading	Science	Conservative
18	1/7/20	Iran stampede: '35 dead' and dozens injured after huge crush at Qassem Soleimani funeral	True	Political/Economy	Neutral
19	1/8/20	Muslim Teen Accused Of Starting Aussie Grass Fire Laughs As He Leaves Court On Tuesday	False/Misleading	Science	Conservative
20	1/9/20	Third busiest abortion facility in Massachusetts could soon shut its doors	True	Political/Economy	Conservative
21	1/13/20	Why Are Volcanoes All Over The Globe Suddenly Shooting Giant Clouds Of Ash Miles Into The Air?	False/Misleading	Science	Neutral
22	1/14/20	Wisconsin Judge Orders Up to 209,000 Listings Purged from Voter Rolls — Finds 3 in Contempt, Orders Fines for Delay	True	Political/Economy	Conservative
23	1/15/20	Bloomberg Draws Paltry Crowd Of 45 At Heavily Advertised Rally	Could Not Determine	Political/Economy	Conservative

24	1/21/20	Pentagon bans Bible verses on dog tags, while Pres. Trump upholds right to pray in public schools	False/Misleading	Political/Economy	Conservative
25	1/22/20	LEAKED FRENCH INTERNAL INTELLIGENCE REPORT CLAIMS 150 NEIGHBORHOODS 'HELD' BY RADICAL ISLAMISTS	No Mode	Political/Economy	Conservative
26	1/23/20	Coronavirus outbreak: China seals off SECOND major city - 18m people on lockdown	True	Science	Neutral
27	1/27/20	Lawmakers Pushing to Make Michigan a 2nd Amendment Sanctuary STATE	True	Political/Economy	Conservative
28	1/28/20	Holy Moses! More Than 175,000 Tickets Requested To See President Trump In New Jersey — Supporters Line Up 48 Hours Early	False/Misleading	Political/Economy	Conservative
29	2/4/20	Ilhan Omar's Dirty Money Hustle Blows Wide Open, Reports Say She Gave 40% Of Her Campaign Spending Went To Loverboy's Firm	False/Misleading	Political/Economy	Conservative
30	2/5/20	DEMS RELEASE ONLY 62% OF IOWA CAUCUS RESULTS — JUST ENOUGH TO HAVE 'MAYOR CHEAT' IN THE LEAD	False/Misleading	Political/Economy	Conservative
31	2/6/20	John Kerry Says That The ENTIRE Obama Admin Was Trying To Get Rid Of The Burisma Prosecutor	No Mode	Political/Economy	Conservative

Table 3: Headlines for Articles Chosen from the Mainstream Conservative News Stream

	Date	Headline	Modal Fact Checker Rating	Topic	Lean of Article
1	11/18/19	Hyundai launches car with a roof-based solar charging system	True	Science	Neutral
2	11/19/19	Pelosi: Trump 's Actions 'Worse' Than Nixon	True	Political/Economy	Neutral
3	11/20/19	Key impeachment witness dodges GOP questions to protect whistleblower	True	Political/Economy	Neutral
4	11/21/19	Smollet Claims He Suffered 'Extreme Emotional Distress' in Malicious Prosecution Lawsuit Against Chicago	True	Human Interest	Neutral
5	12/2/19	'F–K WHITE PEOPLE' graffiti found outside Queens home	True	Human Interest	Neutral
6	12/3/19	Marine veteran turned congressional candidate calls Kaepernick a 'national disgrace'	True	Political/Economy	Conservative
7	12/4/19	Devin Nunes slaps CNN with $435 million defamation lawsuit	True	Political/Economy	Neutral
8	12/5/19	Angry Melania Slams Impeachment Witness for Joking About Son	True	Political/Economy	Conservative
9	12/9/19	Walmart apologizes for sweater featuring Santa with cocaine	True	Human Interest	Neutral
10	12/10/19	Joe Biden Claims No One Told Him About Potential Conflict of Interest With Hunter's Job at Burisma	True	Political/Economy	Conservative
11	12/11/19	House Democrats announce articles of impeachment against Trump: Abuse of power, obstruction of Congress	True	Political/Economy	Neutral
12	12/12/19	Pastors, worship leaders pray for Trump in Oval Office amid impeachment fight	True	Political/Economy	Conservative
13	12/16/19	I was wrong': James Comey admits 'real sloppiness' in Russia probe	True	Political/Economy	Unclear
14	12/17/19	Schiff Says He Would Vote to Impeach Obama If He Engaged in Similar Conduct	True	Political/Economy	Neutral
15	12/18/19	Teen Karol Sanchez staged her own Bronx kidnapping: police sources	True	Human Interest	Neutral

Draft

16	12/19/19	President Trump is impeached in a historic vote by the House, will face trial in the Senate	True	Political/Economy	Neutral
17	1/6/20	Ricky Gervais blasts Hollywood figures as unprincipled, ignorant at Golden Globes	True	Human Interest	Neutral
18	1/7/20	Pelosi Says the House Will Vote on a Resolution to Limit Trump's Military Actions Regarding Iran	True	Political/Economy	Neutral
19	1/8/20	Climate Change? Turns Out Two Dozen Arrested for Setting Australia's Fires	False/Misleading	Science	Conservative
20	1/9/20	Cardi B bashes Trump, says she's seeking Nigerian citizenship amid tensions with Iran	True	Political/Economy	Neutral
21	1/13/20	Bill Gates: My $109 billion net worth shows the economy is not fair	True	Political/Economy	Neutral
22	1/14/20	Trump, first lady cheered at national championship game	True	Political/Economy	Neutral
23	1/15/20	President Trump Gets Thunderous Applause at Clemson and LSU National Championship Game	True	Political/Economy	Conservative
24	1/21/20	Virginia's Capitol flooded with gun rights activists for Second Amendment rally	True	Political/Economy	Conservative
25	1/22/20	CDC confirms first US case of coronavirus that has killed 9 in China	True	Science	Neutral
26	1/23/20	Three US firefighters killed in plane crash while battling wildfires in Australia	True	Science	Neutral
27	1/27/20	Coronavirus may have originated in lab linked to China's biowarfare program	No Mode	Science	Neutral
28	1/28/20	Dershowitz calls out House Dems in Trump's Senate impeachment trial after Bolton shock waves	True	Political/Economy	Conservative
29	2/4/20	Democratic White House Race off to Messy Start as 'Inconsistencies' Delay Iowa Results	True	Political/Economy	Neutral
30	2/5/20	Macy's to close 125 stores, cut 2,000 corporate jobs, in hunt for growth	True	Political/Economy	Neutral

31	2/6/20	Trump acquitted on all charges in Senate impeachment trial	True	Political/Economy	Neutral

Table 4: Headlines for Articles Chosen from the Mainstream Liberal News Stream

	Date	Headline	Modal Fact Checker Rating	Topic	Lean of Article
1	11/18/19	10 shot, four killed at family gathering in Fresno, California	True	Human Interest	Neutral
2	11/19/19	Kanye West calls himself "greatest artist that God has ever created" during Joel Osteen service	True	Human Interest	Neutral
3	11/20/19	Woman Saves Scorched Koala From Bushfire With Shirt Off Her Own Back	True	Science	Neutral
4	11/21/19	Almaas Elman, Somali-Canadian Activist, Is Shot Dead in Mogadishu	True	Political/Economy	Neutral
5	12/2/19	White House will not participate in Wednesday's impeachment hearing	True	Political/Economy	Neutral
6	12/3/19	Duncan Hunter To Plead Guilty In Campaign Finance Case He Called 'Witch Hunt'	True	Political/Economy	Neutral
7	12/4/19	Kamala Harris Dropping Out Of Presidential Race	True	Political/Economy	Neutral
8	12/5/19	'He Showed Us Life': Japanese Doctor Who Brought Water to Afghans Is Killed	True	Human Interest	Neutral
9	12/9/19	Caroll Spinney, legendary 'Sesame Street' puppeteer of Big Bird, dies at 85	True	Human Interest	Neutral
10	12/10/19	Megan Rapinoe is Sports Illustrated's Sportsperson of the Year, only the fourth woman chosen alone	True	Human Interest	Neutral
11	12/11/19	Police Chief Tears Into Ted Cruz, McConnell For Caring More About NRA Than Gun Victims	True	Political/Economy	Neutral
12	12/12/19	Donald Trump Jr killed rare endangered sheep in Mongolia with special permit	True	Other	Liberal
13	12/16/19	Black Women Now Hold Crowns in 5 Major Beauty Pageants	True	Human Interest	Neutral
14	12/17/19	Barack Obama: Women Ruling All Nations Would Improve 'Just About Everything'	True	Political/Economy	Neutral
15	12/18/19	Police investigating whether teen staged her own kidnapping in Bronx	True	Human Interest	Neutral

Draft

16	12/19/19	House impeaches Trump for abuse of power and obstruction in historic rebuke	True	Political/Economy	Neutral
17	1/6/20	Mike Pence Slammed After Falsely Linking Qassem Soleimani To 9/11	True	Political/Economy	Liberal
18	1/7/20	Pentagon Rules Out Striking Iranian Cultural Sites, Contradicting Trump	True	Political/Economy	Liberal
19	1/8/20	All is well,' Trump tweets after Iran targets U.S. forces in missile attack in Iraq	True	Political/Economy	Neutral
20	1/9/20	Ruth Bader Ginsburg says she is cancer-free	True	Political/Economy	Neutral
21	1/13/20	Serena Williams wins first title in 3 years — and donates prize money to Australia wildfire relief	True	Human Interest	Neutral
22	1/14/20	The first Obama-backed documentary receives an Oscar nomination	True	Human Interest	Neutral
23	1/15/20	More than 50 injured after Delta jet dumps fuel on L.A. schools during midair emergency	True	Human Interest	Neutral
24	1/21/20	Katie Sowers Is The First Female And Openly Gay Person To Coach In A Super Bowl	True	Human Interest	Neutral
25	1/22/20	Weather service issues alert for falling iguanas as temperatures drop in Florida	True	Science	Neutral
26	1/23/20	Half of Americans don't know 6m Jews were killed in Holocaust, survey says	True	Political/Economy	Neutral
27	1/27/20	Kobe Bryant's Daughter Gianna, 13, Dead Alongside Father in Calabasas Helicopter Crash	True	Human Interest	Neutral
28	1/28/20	Today really hurts': Families, friends remember those who died in Kobe Bryant crash	True	Human Interest	Neutral
29	2/4/20	State of the Union 2020: Trump addresses nation just before expected acquittal by Senate	True	Political/Economy	Neutral
30	2/5/20	Nancy Pelosi rips up copy of State of the Union speech from Trump	True	Political/Economy	Neutral
31	2/6/20	Kirk Douglas, Hollywood legend and star of Spartacus, dies aged 103	True	Human Interest	Neutral

Table 5: Headlines for Articles Chosen from the Low Quality Unclear News Stream

	Date	Headline	Modal Fact Checker Rating	Topic	Lean of Article
1	11/18/19	Family Facing Jail for Living in RV on Their Own Property to Repair Home After Fire	True	Human Interest	Conservative
2	11/19/19	Shooter Commits Suicide After Being Confronted by Armed Citizen at OK Walmart	False/Misleading	Human Interest	Neutral
3	11/20/19	Pounds lost doesn't mean FAT lost: You CAN lose up to 2 pounds of fat a month – but it takes consistency and patience	False/Misleading	Science	Neutral
4	11/21/19	Ukrainian MP Claims $7.4 Billion Obama-Linked Laundering, Puts Biden Group Take At $16.5 Million	False/Misleading	Political/Economy	Conservative
5	12/2/19	Bestselling Novelist Who Wrote About Vaccine Industry Deception Found Dead	False/Misleading	Human Interest	Unclear
6	12/3/19	Americans Bought Enough Guns on Black Friday to Arm the Marine Corps – Yet Again!	True	Political/Economy	Unclear
7	12/4/19	Ukrainian Neo-Nazis Help Out at Hong Kong Riots, Pan-Democrats Defend Them	Could Not Determine	Political/Economy	Unclear
8	12/5/19	China Repeats US Must Reduce Tariffs For "Phase One" Trade Deal	True	Political/Economy	Neutral
9	12/9/19	Biden Denies Wrongdoing in Ukraine During Testy Interview	True	Political/Economy	Conservative
10	12/10/19	Stressed to the Max? Deep Sleep Can Rewire the Anxious Brain	True	Science	Neutral
11	12/11/19	Since Feeding the Homeless is Illegal, Activists Carry AR-15s to Give Out Food, Supplies	False/Misleading	Political/Economy	Conservative
12	12/12/19	Russia's Only Aircraft Carrier Has Erupted In Flames	True	Political/Economy	Neutral
13	12/16/19	Trump Poised This Week to Become Third U.S. President Impeached	True	Political/Economy	Neutral
14	12/17/19	Kansas City Makes Public Transportation Free, Become The First Major City In The U.S. To Make This Progressive Change	No Mode	Political/Economy	Liberal

15	12/18/19	Wall Street Journal Investigation Finds Amazon.com Selling Dumpster Trash Food & Supplements As New	No Mode	Science	Neutral
16	12/19/19	UN Peacekeepers Fathered Hundreds of Babies With Girls in Haiti as Young as 11	True	Political/Economy	Conservative
17	1/6/20	Senate Republican Eyes Rule Change to Kick Start Trump Impeachment Trial	True	Political/Economy	Neutral
18	1/7/20	Iran Evaluating 13 Retaliation Scenarios To Inflict ”Historic Nightmare” On US	True	Political/Economy	Conservative
19	1/8/20	Key Brain Region Smaller in Birth Control Pill User	True	Science	Neutral
20	1/9/20	The US Military Pollutes More 140 Countries Combined	True	Science	Liberal
21	1/13/20	Alaska man survives three weeks with little food and shelter	True	Human Interest	Neutral
22	1/14/20	Boeing Mocked Lion Air ”Idiots” For Requesting Extra Training For 737 MAX	True	Human Interest	Unclear
23	1/15/20	300 Vultures Occupy Border Patrol Tower, Covering It With “Corrosive” Feces & Vomit	True	Human Interest	Neutral
24	1/21/20	PUNISHING ECONOMY: San Fran’s Democrat tyrants double down on closed businesses, taxing landlords for leaving stores vacant	False/Misleading	Political/Economy	Conservative
25	1/22/20	Another Supposedly Authentic Photo Of A UFO & The Story Behind It	No Mode	Human Interest	Neutral
26	1/23/20	China Quarantines 3rd City As Wuhan Virus Spreads To Singapore	True	Science	Neutral
27	1/27/20	Nature Science Journal Warned About “Pathogens Escaping” Wuhan Level-4 Biosafety Lab (BSL-4) Before Coronavirus Outbreak	False/Misleading	Science	Unclear
28	1/28/20	Death Tolls Rises to 106 as 1,000 Americans Try to Evacuate From Coronavirus-Infected Wuhan	True	Science	Neutral

29	2/4/20	The Coronavirus Was Engineered By Scientists In A Lab Using Well Documented Genetic Engineering Vectors That Leave Behind A "Fingerprint"	False/Misleading	Science	Unclear
30	2/5/20	Earth is About to Enter a 30-Year 'Mini Ice Age' as the Sun Hibernates, Scientist Warns	False/Misleading	Science	Unclear
31	2/6/20	The lies we are being told about the Coronavirus	False/Misleading	Science	Conservative

References

[1] J. Borge-Holthoefer, R. A. Banos, S. Gonzalez-Bailon, and Y. Moreno. Cascading behaviour in complex socio-technical networks. *Journal of Complex Networks*, 1(1):3–24, June 2013.

[2] Ramine Tinati, Leslie Carr, Wendy Hall, and Jonny Bentwood. Identifying communicator roles in twitter. In *Proceedings of the 21st International Conference on World Wide Web*, WWW '12 Companion, pages 1161–1168, New York, NY, USA, April 2012. Association for Computing Machinery.

[3] Haewoon Kwak, Changhyun Lee, Hosung Park, and Sue Moon. What is Twitter, a social network or a news media? In *Proceedings of the 19th International Conference on World Wide Web, WWW '10*, pages 591–600, 2010.

[4] Kevin Aslett, Zeve Sanderson, William Godel, Nathaniel Persily, Jonathan Nagler, Richard Bonneaua, and Joshua A Tucker. An ecologically and externally valid approach to assessing belief in popular misinformation. Working paper, Unpublished.

[5] Hunt Allcott and Matthew Gentzkow. Social media and fake news in the 2016 election. *Journal of economic perspectives*, 31(2):211–36, 2017.

[6] Dan M Kahan. Misconceptions, misinformation, and the logic of identity-protective cognition. *SSRN*, 2017.

[7] Jay J Van Bavel and Andrea Pereira. The partisan brain: An identity-based model of political belief. *Trends in cognitive sciences*, 22(3):213–224, 2018.

[8] Patricia Moravec, Randall Minas, and Alan R Dennis. Fake news on social media: People believe what they want to believe when it makes no sense at all. *MIS Quarterly*, 43, 2019.

[9] Pablo Barberá, John T. Jost, Jonathan Nagler, Joshua A. Tucker, and Richard Bonneau. Tweeting From Left to Right: Is Online Political Communication More Than an Echo Chamber? *Psychological Science*, 26(10):1531–1542, October 2015. Publisher: SAGE Publications Inc.

[10] Pablo Barberá. Birds of the same feather tweet together: Bayesian ideal point estimation using twitter data. *Political Analysis*, 23(1):76–91, 2015.
